# Adverse obstetric outcomes after local treatment for cervical preinvasive and early invasive disease according to cone depth: systematic review and meta-analysis

**DOI:** 10.1136/bmj.i3633

**Published:** 2016-07-28

**Authors:** Maria Kyrgiou, Antonios Athanasiou, Maria Paraskevaidi, Anita Mitra, Ilkka Kalliala, Pierre Martin-Hirsch, Marc Arbyn, Phillip Bennett, Evangelos Paraskevaidis

**Affiliations:** 1 Institute of Reproductive and Developmental Biology, Department of Surgery and Cancer, Faculty of Medicine, Imperial College, London, UK; 2 Queen Charlotte’s and Chelsea-Hammersmith Hospital, Imperial Healthcare NHS Trust, London, UK; 3 University Hospital of Ioannina, Ioannina, Greece; 4 Department of Gynaecological Oncology, Lancashire Teaching Hospitals, Preston, UK; 5 Department of Biophotonics, Lancaster University, Lancaster, UK; 6 Unit of Cancer Epidemiology, Scientific Institute of Public Health, Brussels, Belgium

## Abstract

**Objective** To assess the effect of treatment for cervical intraepithelial neoplasia (CIN) on obstetric outcomes and to correlate this with cone depth and comparison group used.

**Design** Systematic review and meta-analysis.

**Data sources** CENTRAL, Medline, Embase from 1948 to April 2016 were searched for studies assessing obstetric outcomes in women with or without previous local cervical treatment.

**Data extraction and synthesis** Independent reviewers extracted the data and performed quality assessment using the Newcastle-Ottawa criteria. Studies were classified according to method and obstetric endpoint. Pooled risk ratios were calculated with a random effect model and inverse variance. Heterogeneity between studies was assessed with I^2^ statistics.

**Main outcome measures** Obstetric outcomes comprised preterm birth (including spontaneous and threatened), premature rupture of the membranes, chorioamnionitis, mode of delivery, length of labour, induction of delivery, oxytocin use, haemorrhage, analgesia, cervical cerclage, and cervical stenosis. Neonatal outcomes comprised low birth weight, admission to neonatal intensive care, stillbirth, APGAR scores, and perinatal mortality.

**Results** 71 studies were included (6 338 982 participants: 65 082 treated/6 292 563 untreated). Treatment significantly increased the risk of overall (<37 weeks; 10.7% *v* 5.4%; relative risk 1.78, 95% confidence interval 1.60 to 1.98), severe (<32-34 weeks; 3.5% *v* 1.4%; 2.40, 1.92 to 2.99), and extreme (<28-30 weeks; 1.0% *v* 0.3%; 2.54, 1.77 to 3.63) preterm birth. Techniques removing or ablating more tissue were associated with worse outcomes. Relative risks for delivery at <37 weeks were 2.70 (2.14 to 3.40) for cold knife conisation, 2.11 (1.26 to 3.54) for laser conisation, 2.02 (1.60 to 2.55) for excision not otherwise specified, 1.56 (1.36 to 1.79) for large loop excision of the transformation zone, and 1.46 (1.27 to 1.66) for ablation not otherwise specified. Compared with no treatment, the risk of preterm birth was higher in women who had undergone more than one treatment (13.2% *v* 4.1%; 3.78, 2.65 to 5.39) and with increasing cone depth (≤10-12 mm; 7.1% *v* 3.4%; 1.54, 1.09 to 2.18; ≥10-12 mm: 9.8% *v* 3.4%, 1.93, 1.62 to 2.31; ≥15-17 mm: 10.1% *v* 3.4%; 2.77, 1.95 to 3.93; ≥20 mm: 10.2% *v* 3.4%; 4.91, 2.06 to 11.68). The choice of comparison group affected the magnitude of effect. This was higher for external comparators, followed by internal comparators, and ultimately women with disease who did not undergo treatment. In women with untreated CIN and in pregnancies before treatment, the risk of preterm birth was higher than the risk in the general population (5.9% *v* 5.6%; 1.24, 1.14 to 1.35). Spontaneous preterm birth, premature rupture of the membranes, chorioamnionitis, low birth weight, admission to neonatal intensive care, and perinatal mortality were also significantly increased after treatment.****

**Conclusions** Women with CIN have a higher baseline risk for prematurity. Excisional and ablative treatment further increases that risk. The frequency and severity of adverse sequelae increases with increasing cone depth and is higher for excision than for ablation.

## Introduction

The mean age of women undergoing local treatment for cervical preinvasive cervical disease (cervical intraepithelial neoplasia or CIN) is similar to the age of women having their first child. Local cervical treatment has been correlated to an increased risk of preterm birth, perinatal morbidity, and mortality in a subsequent pregnancy.^1^ ^2^ ^3^ ^4^ ^5^ ^6^ The underlying mechanism is unclear; hypotheses include immunomodulation relating to infection with human papillomavirus (HPV) affecting parturition pathways and acquired “mechanical weakness” secondary to loss of cervical tissue.^7^ ^8^

In England alone in 2013-14, about 3.6 million women aged 25-64 attended for cervical screening, and over 23 800 cervical procedures were carried out,^9^ nearly all in an outpatient setting. In contrast, in the United States there are about 400 000 cases of preinvasive disease a year.^10^ The regulations in colposcopy are more liberal, leading to wide variation in clinical practice. In Germany, treatment for CIN is still commonly performed with the cold knife under general analgesia.^11^ The long term sequelae of treatment therefore remain an important international issue to healthcare professionals and women, whatever the clinical setting.

Since the first systematic review almost a decade ago on reproductive risk associated with treatment^1^ more than 50 observational studies have been published confirming^12^ ^13^ or disputing these associations^14^ ^15^; some of these reported data from large population based datasets. Individual attempts to synthesise parts of this rapidly evolving evidence base in small systematic reviews and meta-analyses reached contradictory conclusions^1^ ^2^ ^3^ ^4^ ^16^ ^17^ ^18^ ^19^ and initiated debates and confusion within the scientific community.^2^ ^16^ ^17^ ^18^ ^19^ Whether these discrepancies were due to questionable quality of some of these primary and secondary studies or to differences in the explored comparisons,^4^ ^16^ ^17^ ^18^ the subject is open to a definitive comprehensive high quality synthesis of the existing evidence that will be highly informative to women, clinicians, and policy makers.

Media publicity has heightened public awareness that treatment for cervical precancer is associated with increased reproductive morbidity. There has been a substantial increase in inquiries from patients and clinicians on the risks associated with different treatment techniques and cone depths^20^ ^21^ and as to how this risk can be managed and prevented. With a rapidly evolving evidence base and lack of a robust synthesis of the published literature, these questions are becoming increasingly difficult to answer.

We carried out a systematic review and meta-analysis to explore the impact of treatment for cervical preinvasive and early invasive disease on obstetric outcomes and how this risk could be modified by the cone depth and comparison group.

## Methods

### Inclusion criteria and outcomes

We included all studies that reported on obstetric outcomes (over 24 weeks’ gestation) in women who had previously received local cervical treatment for CIN or early invasive cervical cancer compared with outcomes in women with no history of treatment. Studies reporting on the outcomes after two or more treatments were also included. The interventions included any type of treatment: excisional (cold knife conisation; laser conisation; needle excision of the transformation zone, also known as straight wire excision; large loop excision of the transformation zone, also known as loop electrosurgical excisional procedure) or ablative (laser ablation; radical diathermy; cold coagulation; and cryotherapy). In studies that reported on the impact of several techniques, when possible we extracted data for each specific method. If the outcomes were not reported separately for each technique, we analysed the intervention under broader terms—that is, excisional treatment not otherwise specified, ablative treatment not otherwise specified, and treatment not otherwise specified.

Women were included irrespective of the grade of the lesion for both squamous and glandular intraepithelial neoplasia. We excluded studies that did not include an untreated reference population, compared different treatment techniques without an untreated control, or compared outcomes for treatments performed during pregnancy.

Studies were included irrespective of the type of untreated reference population that could have been drawn from one of the following sources: external group from general population that was mostly matched or adjusted for confounders; internal group with self matching of the pregnancies for the same women before and after treatment; internal group of women who had also delivered before treatment; women undergoing colposcopy with or without CIN/biopsy but no treatment; women with high grade disease but no treatment (high grade squamous intraepithelial lesion).

We assessed obstetric outcomes of pregnancies progressing beyond 24 weeks’ gestation. We examined both maternal and neonatal outcomes. The maternal outcomes included overall (<37 weeks’ gestation), severe (<32-34 weeks), and extreme (<28-30 weeks) prematurity (all preterm birth, iatrogenic and spontaneous). We also assessed preterm birth in singleton and multiple pregnancies, in nulliparous and parous women, for single and repeat cones, for different cone depths and volumes, and for different comparison groups. We further assessed other maternal outcomes that included: overall (<37 weeks’ gestation), severe (<32-34), and extreme (<28-30)****spontaneous prematurity—that is, non-iatrogenic); threatened preterm birth; premature rupture of the membranes; chorioamnionitis; mode of delivery (caesarean section, instrumental deliveries); length of labour (precipitous, prolonged); induction of labour or use of oxytocin; haemorrhage (antepartum, postpartum); analgesia (epidural, pethidine, not otherwise specified); cervical stenosis; and cervical cerclage. The neonatal outcomes included: low birth weight (<2500 g, <2000 g, <1500 g, and <1000 g), admission to neonatal intensive unit, perinatal mortality, stillbirth, and Apgar score.

When there was heterogeneity in the cut offs used in different studies for cone depth and classification of prematurity, we grouped these together when possible (that is, 32-34 weeks included both cut offs; 10-12 mm cone depth included studies grouping at both these cut offs with and without the values equal to these numbers).

### Literature search, data extraction, and risk of bias

We searched three electronic databases (CENTRAL, Medline, and Embase) and targeted reports published between 1948 and April 2016. We used keywords including “cervical intraepithelial neoplasia (CIN)”, “cervical cancer”, “LLETZ or LEEP”, “conisation”, “excision”, “pregnancy”, “obstetric”, “preterm birth,” and “prematurity”. The full strategy is included in appendix 1. In an attempt to identify any articles missed by the initial search or any unpublished data, we hand searched the references of the retrieved articles and meta-analyses and the proceedings of relevant conferences. There was no language restriction.

From each study, we extracted data on the study design and setting, the study population, the interventions examined, the comparison group, the quality of the data and risk of bias, and the outcomes assessed. From each study and for each outcome we retrieved the number of events in treated and untreated women. If necessary, we contacted authors to obtain additional data if the numbers provided in the published report did not allow sufficient precision in the data extraction.

We used the Newcastle-Ottawa score to formally assess the quality of non-randomised cohort studies,^22^ according to the MOOSE checklist.^23^ This scoring system assesses the cohort selection, comparability, and assessment of outcomes to give a maximum score of 9 (highest quality).

Two investigators (MK, AA) independently performed the literature search, assessed the eligibility and quality of the retrieved papers, and performed the data extraction. The two authors then compared the results and disagreements were resolved by discussion. If required, consensus was reached with the involvement of a third investigator (MA).

### Data synthesis and assessment of heterogeneity

We calculated the risk ratios and 95% confidence intervals for each reported outcome in the treated versus untreated women for dichotomous outcomes using Cochrane Revman 5 software. We used a random effect model and inverse variance weighting for all meta-analyses.^24^ In studies with multiple treatment groups, we proportionally divided the “shared” comparison group into the number of treatment groups; we treated comparisons between each treatment group and the split comparison group as independent comparisons. If a study presented data for more than one comparison group, we used the external comparison group of women with or without disease in preference to internal controls. If data were not of suitable quality for meta-analysis, we reported the results as a narrative in the text of the review.

We assessed heterogeneity between studies with the Cochran Q test, visual inspection of forest plots,^25^ estimation of the percentage of heterogeneity between studies that cannot be ascribed to sampling variation (I^2^ statistic),^26^ and a formal test of the significance for heterogeneity.^27^ If there was evidence of substantial heterogeneity, the possible reasons for this were investigated and reported.

We performed a series of subgroup analyses. We analysed the data separately for each treatment, in groups of ablative and excisional techniques, and as a whole irrespective of the type of method used. We further analysed the data according to the cone depth. Given the non-randomised nature of the included studies, we assessed whether the choice of comparison group affected the risk estimate for each outcome and overinflated the effect of treatment that could be partly attributed to other confounders. We therefore distinguished the different untreated comparison groups used across studies and performed subgroup analyses for the risk of preterm birth for each individual comparator (external; internal (self matching); internal (pregnancies before treatment); colposcopy but no treatment; high grade squamous intraepithelial lesion but no treatment). Furthermore, we performed sensitivity analysis to assess the impact of the quality of the studies on some selected outcomes. We calculated the median score from the Newcastle-Ottawa scale and performed sensitivity analysis for studies that scored more than the median. We performed subgroup analyses based on the cohort selection in the Newcastle-Ottawa score (truly or somewhat representative) and the comparability of the groups (those with scores of 1 or 2). Finally, we performed meta-regression analysis to assess the impact of some factors on the risk of preterm birth (<37 weeks). These included the quality of the studies (based on the Newcastle-Ottawa score); year of study (1979-89, 1990-99, 2000-09, 2010-15); type of treatment (excision or ablation); type of comparator (external, internal-pregnancies before treatment, internal-self matching, CIN but no treatment, high grade squamous intraepithelial lesion but no treatment).

### Patient involvement

Patients and the wider public were involved from the outset through informal interviews in the clinic and through patient advocate representative bodies. The research questions and outcomes were developed based on the patients’ concerns and priorities. Patients were not involved in the interpretation of results or writing of the article. The results will be disseminated to the lay audience through the authors’ involvement with charities and through public presentations.

## Results

We identified 406 potentially eligible studies that fulfilled the inclusion criteria of this review.^5^ ^12^ ^13^ ^14^ ^15^ ^28^ ^29^ ^30^ ^31^ ^32^ ^33^ ^34^ ^35^ ^36^ ^37^ ^38^ ^39^ ^40^ ^41^ ^42^ ^43^ ^44^ ^45^ ^46^ ^47^ ^48^ ^49^ ^50^ ^51^ ^52^ ^53^ ^54^ ^55^ ^56^ ^57^ ^58^ ^59^ ^60^ ^61^ ^62^ ^63^ ^64^ ^65^ ^66^ ^67^ ^68^ ^69^ ^70^ ^71^ ^72^ ^73^ ^74^ ^75^ ^76^ ^77^ ^78^ ^79^ ^80^ ^81^ ^82^ ^83^ ^84^ ^85^ ^86^ ^87^ ^88^ ^89^ ^90^ ^91^ ^92^ ^93^ No unpublished studies were identified. We excluded studies without an untreated reference population,^94^ ^95^ ^96^ ^97^ ^98^ ^99^ ^100^ ^101^ ^102^ ^103^ ^104^ ^105^ ^106^ ^107^ ^108^ ^109^ ^110^ ^111^ ^112^ ^113^ ^114^ ^115^ ^116^ ^117^ ^118^ ^119^ studies that included women treated during pregnancy,^120^ ^121^ studies assessing fertility and early pregnancy outcomes below 24 weeks’ gestation,^122^ ^123^ ^124^ ^125^ ^126^ ^127^ studies assessing outcomes after treatment in high risk populations,^128^ ^129^ and studies assessing the impact of CIN on outcomes without information as to whether treatment was performed.^130^ ^131^ ^132^Figure 1 shows more details of the literature search and the reasons for exclusion.^133^

**Figure f1:**
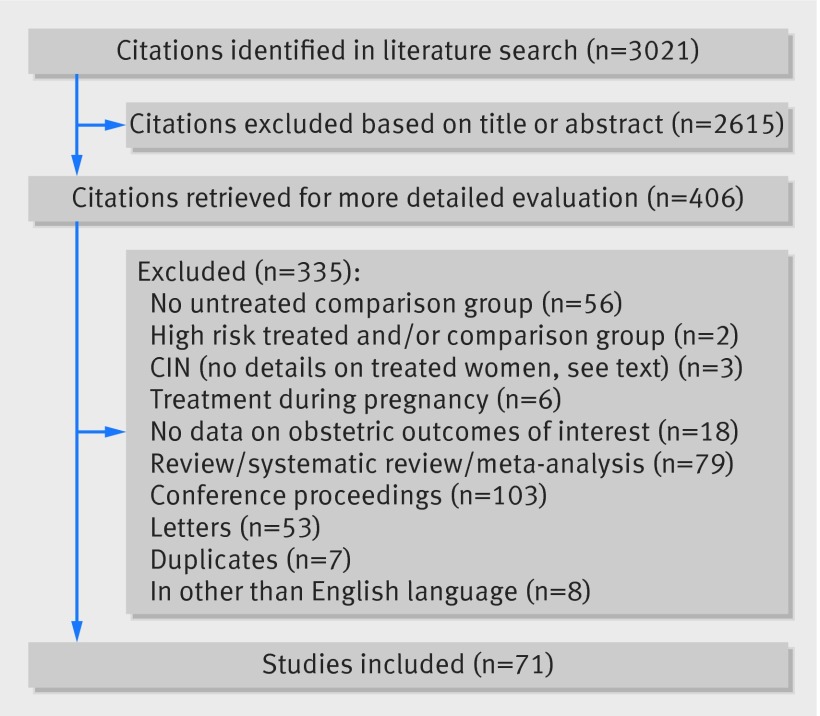
**Fig 1** Identification of studies to include in analysis of adverse obstetric outcomes after local treatment for cervical preinvasive and early invasive disease

Table A in appendix 2 shows detailed characteristics of the included studies and the outcomes examined. Most studies were retrospective, with only five prospective reports.^71^ ^77^ ^78^ ^80^ ^82^ All were cohort studies, apart from one case-control study by Castanon and colleagues.^85^ There were no randomised controlled studies. Fourteen studies examined the impact of cold knife conisation,^13^ ^28^ ^29^ ^30^ ^32^ ^33^ ^34^ ^37^ ^60^ ^61^ ^62^ ^82^ ^87^ ^89^ 10 studied laser conisation,^42^ ^46^ ^47^ ^48^ ^49^ ^51^ ^52^ ^56^ ^76^ ^78^ one studied needle excision of the transformation zone.^13^ 34 studied large loop excision of the transformation zone,^13^ ^39^ ^40^ ^41^ ^44^ ^45^ ^50^ ^55^ ^56^ ^57^ ^58^ ^59^ ^60^ ^62^ ^63^ ^65^ ^66^ ^67^ ^68^ ^69^ ^73^ ^74^ ^76^ ^77^ ^78^ ^80^ ^81^ ^82^ ^83^ ^86^ ^87^ ^88^ ^90^ ^91^ eight studied laser ablation,^35^ ^38^ ^39^ ^47^ ^49^ ^54^ ^56^ ^62^ one studied radical diathermy,^62^ two studied cryotherapy,^31^ ^60^ 16 studied excision not otherwise specified,^5^ ^12^ ^14^ ^15^ ^53^ ^64^ ^70^ ^71^ ^72^ ^75^ ^78^ ^79^ ^84^ ^85^ ^90^ ^93^ five studied ablation not otherwise specified,^12^ ^14^ ^53^ ^70^ ^87^ and three studied treatment not otherwise specified.^36^ ^43^ ^92^ There were five types of untreated comparison groups. Some used an external comparator,^5^ ^12^ ^13^ ^14^ ^15^ ^28^ ^29^ ^33^ ^35^ ^36^ ^37^ ^38^ ^39^ ^40^ ^41^ ^42^ ^43^ ^44^ ^45^ ^48^ ^49^ ^51^ ^52^ ^53^ ^54^ ^55^ ^57^ ^58^ ^59^ ^60^ ^61^ ^64^ ^65^ ^66^ ^67^ ^68^ ^69^ ^70^ ^71^ ^72^ ^73^ ^74^ ^75^ ^76^ ^77^ ^78^ ^79^ ^80^ ^81^ ^83^ ^86^ ^87^ ^89^ ^92^ ^93^ others compared with the pregnancies before treatment in the treated population (internal)^5^ ^15^ ^30^ ^31^ ^32^ ^34^ ^45^ ^46^ ^47^ ^58^ ^73^ ^74^ ^84^ ^91^ or used self matching for women who delivered both before and after treatment (internal),^13^ ^15^ ^43^ ^48^ ^51^ ^64^ ^66^ some compared with women who underwent colposcopy with or without CIN and/or biopsy but had no treatment,^15^ ^56^ ^62^ ^63^ ^67^ ^68^ ^77^ ^81^ ^82^ ^84^ ^88^ ^90^ ^91^ ^93^ and some with women with high grade disease but no treatment.^13^ ^53^ ^70^ All studies that used an external comparison group either matched for known risk factors or performed regression analysis to control for known confounders; four studies did not control for any confounders.^43^ ^61^ ^65^ ^76^

Table B in appendix 2 provides more details on the quality assessment for observational studies with the Newcastle-Ottawa score. Most studies scored 8 or 9 points, 10 scored 7,^30^ ^35^ ^43^ ^45^ ^46^ ^47^ ^50^ ^61^ ^72^ ^76^ and two scored 6.^38^ ^65^

### Maternal outcomes 

The risk of preterm birth was significantly increased after cervical treatment (table 1). For all treatment types, this was the case for overall prematurity at less than 37 weeks’ gestation (relative risk 1.78, 95% confidence interval 1.60 to 1.98), for severe prematurity less than 32-34 weeks’ gestation (2.40, 1.92 to 2.99), and extreme prematurity less than 28-30 weeks’ gestation (2.54, 1.77 to 3.63) (table 1). Figure 2 shows the risk associated with LLETZ versus no treatment. The forest plot in appendix 3 shows the risks for all treatment techniques versus no treatment. The magnitude of the effect of treatment was higher for more radical treatment techniques and for excision rather than ablation. More specifically, the risk of preterm birth at less than 37 weeks’ gestation was higher for cold knife conisation (2.70, 2.14 to 3.40), laser conisation (2.11, 1.26 to 3.54), excision not otherwise specified (2.02, 1.60 to 2.55), large loop excision of the transformation zone (1.56, 1.36 to 1.79), and ablation not otherwise specified (1.46, 1.27 to 1.66). Similar trends were noted for severe and extreme prematurity.

**Table 1 tbl1:** Preterm birth in women with cervical intraepithelial neoplasia (CIN) for treated versus untreated women*

Preterm birth	No of studies	Total No of women	No (%) of women		Effect estimate RR (95% CI)	P value for heterogeneity (I^2^%)
			Treated	Untreated		
**<37 weeks’ gestation**						
All treatment types	60	5 244 560	6506/60 619 (10.7)	281 575/5 183 941 (5.4)	1.78 (1.60 to 1.98)	<0.001 (88)
CKC	12	39 102	126/844 (14.9)	2321/38 258 (6.1)	2.70 (2.14 to 3.40)	0.62 (0)
LC	9	1464	96/672 (14.3)	58/792 (7.3)	2.11 (1.24 to 3.57)	0.02 (56)
NETZ	1	7399	17/71 (23.9)	301/7328 (4.1)	5.83 (3.80 to 8.95)	N/E
LLETZ	26	1 445 341	1724/21 318 (8.1)	66 607/1 424 023 (4.7)	1.56 (1.36 to 1.79)	<0.001 (69)
LA	7	4710	168/1867 (9.0)	242/2843 (8.5)	1.04 (0.86 to 1.26)	0.48 (0)
CT	2	238	4/151 (2.6)	2/87 (2.3)	1.02 (0.22 to 4.77)	0.67 (0)
RD	1	2150	109/760 (14.3)	123/1390 (8.8)	1.62 (1.27 to 2.06)	N/E
Excisional treatment NOS	15	3 107 438	3788/28 104 (13.4)	183 133/3 079 334 (5.9)	2.02 (1.60 to 2.55)	<0.001 (95)
Ablative treatment NOS	5	595 272	430/6482 (6.6)	26 804/588 790 (4.6)	1.46 (1.27 to 1.66)	0.22 (30)
Treatment NOS	3	41 401	44/350 (12.6)	1979/41 051 (4.8)	2.20 (1.28 to 3.78)	0.07 (62)
**<32-34 weeks’ gestation**						
All treatment types	25	3 795 351	1375/39 647 (3.5)	53 835/3 755 704 (1.4)	2.40 (1.92 to 2.99)	<0.001 (82)
CKC	5	36 979	15/283 (5.3)	920/36 696 (2.5)	3.07 (1.72 to 5.49)	0.65 (0)
NETZ	1	7399	5/71 (7.0)	49/7328 (0.7)	10.53 (4.33 to 25.65)	N/E
LLETZ	11	791 554	237/11 569 (2.0)	9504/779 985 (1.2)	2.13 (1.66 to 2.75)	0.08 (40)
CT	1	58	1/36 (2.8)	0/22 (0.0)	1.86 (0.08 to 43.87)	N/E
Excisional treatment NOS	10	2 832 112	1000/22 562 (4.4)	42 598/2 809 550 (1.5)	3.05 (1.95 to 4.78)	<0.001 (91)
Ablative treatment NOS	2	120 762	26/2549 (1.0)	686/118 213 (0.6)	1.59 (1.08 to 2.35)	0.92 (0)
Treatment NOS	2	6487	91/2577 (3.5)	78/3910 (2.0)	1.65 (1.13 to 2.42)	0.25 (24)
**<28-30 weeks’ gestation**						
All treatment types	9	3 912 106	403/39 154 (1.0)	12 887/3 872 952 (0.3)	2.54 (1.77 to 3.63)	<0.001 (81)
CKC	2	7118	2/150 (1.3)	19/6968 (0.3)	4.52 (0.83 to 24.54)	0.74 (0)
NETZ	1	7399	3/71 (4.2)	21/7328 (0.3)	14.74 (4.50 to 48.32)	N/E
LLETZ	3	502 778	59/8899 (0.7)	1224/493 879 (0.2)	2.57 (1.97 to 3.35)	0.9 (0)
Excisional treatment NOS	4	2 821 185	287/21 984 (1.3)	9854/2 799 201 (0.4)	2.90 (1.52 to 5.52)	<0.001 (88)
Ablative treatment NOS	3	568 217	23/6125 (0.4)	1739/562 092 (0.3)	1.38 (0.81 to 2.36)	0.21 (35)
Treatment NOS	1	5409	29/1925	30/3484	1.75 (1.05 to 2.91)	N/E

CKC=cold knife conisation; CT=cryotherapy; LA=laser ablation; LC=laser conisation; LLETZ=large loop excision of transformation zone; N/E=not eligible; NETZ=needle excision of transformation zone; NOS=not otherwise specified; RD=radical diathermy.

*If study had more than one comparison groups, we used external groups (external general, external untreated women who had colposcopy+/-CIN+/-biopsy, women with HSIL but no treatment) in preference to internal comparators (self matching or pregnancies before treatment).

†In cases of heterogeneity in cut-offs used for classification of prematurity, these were grouped together when possible (for instance, 32-34 or 28-30 weeks included both cut offs).

**Figure f2:**
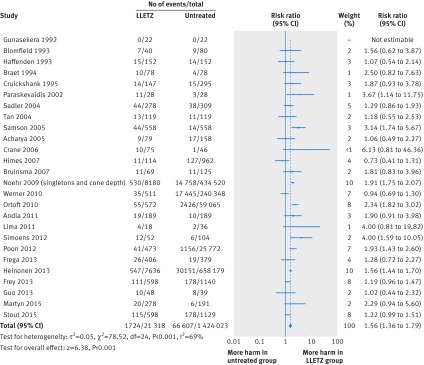
**Fig 2** Meta-analysis of studies on preterm birth (<37 weeks) in women treated with large loop excision of transformation zone versus untreated women

Treatment was also associated with an increased risk of preterm birth for women with multiple pregnancies for some but not all treatments (table C, appendix 2). Have asked author, but the results were inconsistent because of the small number of studies. The impact of treatment was no different for nulliparous and multiparous women (data not shown). The effect of multiple as opposed to single treatments on the risk of prematurity was substantially higher in comparisons with untreated women (relative risk 3.78 (95% confidence interval 2.65 to 5.39) for repeated treatment and 1.75 (1.49 to 2.06) for single treatment, table 2). Compared with no treatment, the relative risk of preterm birth for two excisional treatments not otherwise specified was as high as 5.48 (2.68 to 11.24) and that of two loop excisions as high as 2.81 (2.33 to 3.39).

**Table 2 tbl2:** Preterm birth (<37 weeks’ gestation) in women with cervical intraepithelial neoplasia (CIN) for treated versus untreated women according to number of treatments*

	No of studies	Total No of women	No (%) of women		Effect estimate RR (95% CI)	P value for heterogeneity (I^2^%)
			Treated	Untreated		
**Single treatment**						
All treatment types	17	1 367 023	1519/20 302 (7.5)	56 185/1 346 721 (4.2)	1.75 (1.49 to 2.06)	<0.001 (79)
CKC	3	36 783	38/179 (21.2)	2250/36604 (6.1)	2.89 (2.08 to 4.03)	0.42 (0)
LC	2	657	34/335 (10.1)	29/322 (9.0)	1.06 (0.54 to 2.09)	0.17 (48)
NETZ	1	7399	17/71 (23.9)	301/7328 (4.1)	5.83 (3.80 to 8.95)	N/E
LLETZ	9	1 277 874	1139/16 755 (6.8)	51 075/1 261 119 (4.0)	1.74 (1.45 to 2.10)	<0.001 (75)
LA	4	1421	58/624 (9.3)	68/797 (8.5)	1.07 (0.66 to 1.74)	0.17 (40)
Excisional treatment NOS	3	32 106	197/1816 (10.8)	1840/30 290 (6.1)	1.88 (1.20 to 2.93)	0.1 (57)
Ablative treatment NOS	1	10 783	36/522 (6.9)	622/10 261 (6.1)	1.14 (0.82 to 1.57)	N/E
**Repeat treatment**						
All treatment types	11	1 317 284	191/1442 (13.2)	54 142/1 315 842 (4.1)	3.78 (2.65 to 5.39)	<0.001 (75)
CKC/LA	1	99	2/2 (100.0)	6/97 (6.2)	12.56 (5.11 to 30.87)	N/E
LC/LC	1	270	6/20 (30.0)	20/250 (8.0)	3.75 (1.70 to 8.27)	N/E
LLETZ/LLETZ	4	1 202 174	139/1195 (11.6)	48 586/1 200 979 (4.0)	2.81 (2.33 to 3.39)	0.35 (9)
LLETZ/treatment NOS	1	298	9/41 (22.0)	6/257 (2.3)	9.40(3.53 to 25.03)	N/E
Excisional NOS/excisional treatment NOS	3	73 651	17/57 (29.8)	3034/73 594 (4.1)	5.48 (2.68 to 11.24)	0.16 (45)
Treatment NOS/treatment NOS	2	40 792	18/127 (14.2)	2490/40 665 (6.1)	1.71 (1.10 to 2.67)	0.85 (0)

CKC=cold knife conisation; CT=cryotherapy; LA=laser ablation; LC=laser conisation; LLETZ=large loop excision of transformation zone; N/E=not eligible; NETZ=needle excision of transformation zone; NOS=not otherwise specified; RD=radical diathermy.

*If study had more than one comparison groups, we used external groups (external general, external untreated women that had colposcopy+/−CIN+/−biopsy, women with HSIL but no treatment) in preference to internal comparators (self matching or pregnancies before treatment).

The analysis of the risk according to the cone dimensions showed that the risk increases progressively with increasing cone depth (table 3; fig 3) or cone volume (table 3). The risk for treated versus untreated women was significantly higher for women with cone depth ≤10-12 mm (relative risk 1.54, 95% confidence interval 1.09 to 2.18). The magnitude of effect increased with increasing cone depth (1.93 (1.62 to 2.31) for ≥10-12 mm, 2.77 (1.95 to 3.93) for ≥15-17 mm, and 4.91 (2.06 to 11.68) for ≥20 mm; table 3). The trend was similar with increasing cone volume (2.25 (1.09 to 4.66) for ≤6 cc and 13.9 (5.09 to 37.98) for ≥6 cc; table 3). Further analyses of the individual cone depth cut offs not grouped together showed similar results (data not shown).

**Table 3 tbl3:** Preterm birth (<37 weeks’ gestation) in women with cervical intraepithelial neoplasia (CIN) for treated versus untreated women according to cone depth and volume

	No of studies	Total No of women	No (%) of women		Effect estimate RR (95% CI)	P value for heterogeneity (I^2^%)
			Treated	Untreated		
**Cone depth**						
**≤10-12 mm**						
All treatment types	8	550 929	293/4105 (7.1)	18 720/546 824 (3.4)	1.54 (1.09 to 2.18)	0.004 (67)
LC	1	105	1/41 (2.4)	3/64 (4.7)	0.52 (0.06 to 4.83)	N/E
LLETZ	3	544 907	98/1600 (6.1)	18 448/543 307 (3.4)	2.01 (1.28 to 3.15)	0.13 (51)
Excisional treatment NOS	4	5917	194/2464 (7.9)	269/3453 (7.8)	1.20 (0.78 to 1.85)	0.15 (44)
**≥10-12 mm**						
All treatment types	8	552 711	571/5845 (9.8)	18 723/546 866 (3.4)	1.93 (1.62 to 2.31)	0.13 (37)
LC	1	87	5/23 (21.7)	3/64 (4.7)	4.64 (1.20 to 17.88)	N/E
LLETZ	3	546 134	193/2827 (6.8)	18 448/543 307 (3.4)	2.29 (1.57 to 3.34)	0.2 (37.23)
Excisional treatment NOS	4	6490	373/2995 (12.5)	272/3495 (7.8)	1.68 (1.41 to 1.99)	0.37 (5.32)
**≤15-17 mm**						
All treatment types	4	545 939	149/2614 (5.7)	18 493/543 325 (3.4)	1.36 (1.15 to 1.61)	0.61 (0)
LC	1	164	0/14 (0.0)	7/150 (4.7)	0.67 (0.04 to 11.18)	N/E
LLETZ	2	545 119	117/2370 (4.9)	18 434/542 749 (3.4)	1.42 (1.18 to 1.70)	0.41 (0)
Excisional treatment NOS	1	656	32/230 (13.9)	52/426 (12.2)	1.14 (0.76 to 1.72)	N/E
**≥15-17 mm**						
All treatment types	4	544 986	167/1661 (10.1)	18 493/543 325 (3.4)	2.77 (1.95 to 3.93)	0.1 (53)
LC	1	211	14/61 (23.0)	7/150 (4.7)	4.92 (2.09 to 11.59)	N/E
LLETZ	2	544 248	128/1499 (8.5)	18 434/542 749 (3.4)	3.16 (1.54 to 6.48)	0.08 (67)
Excisional treatment NOS	1	527	25/101 (24.8)	52/426 (12.2)	2.03 (1.33 to 3.10)	N/E
**≤20 mm**						
All treatment types	3	545 992	174/3093 (5.6)	18 441/542 899 (3.4)	1.60 (1.38 to 1.87)	0.62 (0)
LC	1	183	2/33 (6.1)	7/150 (4.7)	1.30 (0.28 to 5.97)	N/E
LLETZ	2	545 809	172/3060 (5.6)	18 434/542 749 (3.4)	1.61 (1.38 to 1.87)	0.35 (0)
**≥20 mm**						
All treatment types	3	543 750	87/851 (10.2)	18441/542 899 (3.4)	4.91 (2.06 to 11.68)	0.01 (77)
LC	1	192	12/42 (28.6)	7/150 (4.7)	6.12 (2.57 to 14.57)	N/E
LLETZ	2	543 558	75/809 (9.3)	18 434/542 749 (3.4)	4.72 (1.25 to 17.80)	0.01 (83)
**10-13 to 15-16 mm**						
All treatment types	3	544 534	75/1359 (5.5)	18 486/543 175 (3.4)	1.32 (1.04 to 1.66)	0.82 (0)
LLETZ	2	543 994	57/1245 (4.6)	18 434/542 749 (3.4)	1.32 (1.02 to 1.72)	0.53 (0)
Excisional treatment NOS	1	540	18/114 (15.8)	52/426 (12.2)	1.29 (0.79 to 2.12)	N/E
**15-16 to 19-20 mm**						
All treatment types	3	543 608	55/709 (7.8)	18441/542 899 (3.4)	2.24 (1.73 to 2.91)	0.42 (0)
LC	1	169	2/19 (10.5)	7/150 (4.7)	2.26 (0.50 to 10.08)	N/E
LLETZ	2	543 439	53/690 (7.7)	18 434/542 749 (3.4)	2.53 (1.42 to 4.51)	0.19 (43)
**Cone volume**						
**<3 cc**						
All treatment types	1	496	16/218 (7.3)	10/278 (3.6)	2.04 (0.94 to 4.41)	N/E
LLETZ	1	496	16/218 (7.3)	10/278 (3.6)	2.04 (0.94 to 4.41)	N/E
**>3 cc**						
All treatment types	1	338	9/60 (15.0)	10/278 (3.6)	4.17 (1.77 to 9.82)	N/E
LLETZ	1	338	9/60 (15.0)	10/278 (3.6)	4.17 (1.77 to 9.82)	N/E
**<6 cc**						
All treatment types	1	550	22/272 (8.1)	10/278 (3.6)	2.25 (1.09 to 4.66)	N/E
LLETZ	1	550	22/272 (8.1)	10/278 (3.6)	2.25 (1.09 to 4.66)	N/E
**>6 cc**						
All treatment types	1	284	3/6 (50.0)	10/278 (3.6)	13.9 (5.09 to 37.98)	N/E
LLETZ	1	284	3/6 (50.0)	10/278 (3.6)	13.9 (5.09 to 37.98)	N/E
**3-6 cc**						
All treatment types	1	332	6/54 (11.1)	10/278 (3.6)	3.09 (1.17 to 8.14)	N/E
LLETZ	1	332	6/54 (11.1)	10/278 (3.6)	3.09 (1.17 to 8.14)	N/E

LC=laser conisation; LLETZ=large loop excision of transformation zone; N/E=not eligible; NETZ=needle excision of transformation zone; NOS=not otherwise specified.

*In cases of heterogeneity in cut offs used for classification of cone depth, these were grouped together when possible (for instance, 10-12 mm in depth included studies using either cut off ≥11-12 or ≤11-12 mm as some studies included depths equal to cut off and others did not).

**Figure f3:**
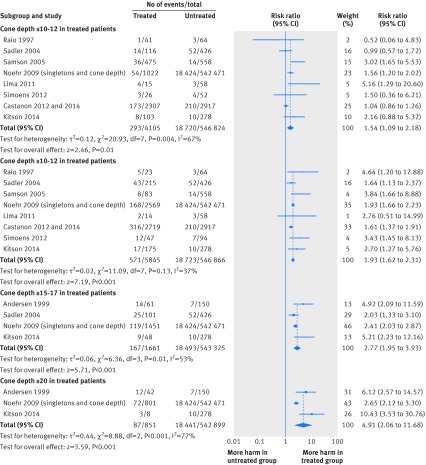
**Fig 3** Preterm birth (<37 weeks) in women treated for CIN according to cone depth (≤10-12 mm, ≥10-12 mm, ≥15-17 mm, ≥20 mm) versus untreated women

The comparison of women treated with different cone depths showed that deeper excisions significantly increased the risk of preterm birth compared with less deep excisions, and the magnitude of the effect increased in deeper cones. The relative risk were 1.54 (95% confidence interval 1.31 to 1.80) for ≥10-12 mm *v* ≤10-12 mm, 1.82 (1.47 to 2.26) for ≥15-17 mm *v* ≤15-17 mm, and 1.82 (1.47 to 2.26) for ≥20 mm *v* ≤20 mm (fig 4). Full data are also provided in table D, appendix 2. The findings were similar for the comparison of cone volumes (2.04 (0.95 to 4.39) for ≥3-4 cc *v* ≤3-4 cc (15.0% *v* 7.3%, one study, 278 women); 6.18 (2.53 to 15.13) for ≥6 cc *v* ≤6 cc (50.0% *v* 8.1%, one study, 278 women).

**Figure f4:**
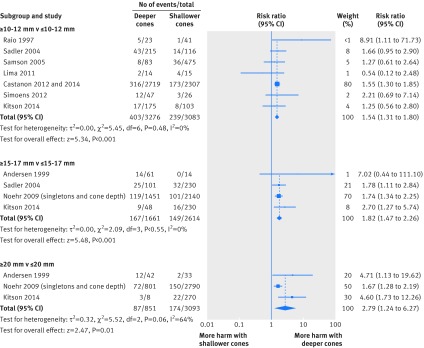
**Fig 4** Preterm birth (<37 weeks’ gestation) in women treated for CIN according to cone depth

We assessed the impact that the choice of comparison group can have on the magnitude of effect in a subgroup analysis that classified different studies according to the comparator used (table 4). The results suggested that treatment significantly increased the risk of preterm birth at less than 37 weeks’ gestation irrespective of the comparison group used. The magnitude of effect was higher when an external comparison group was used (relative risk 1.93, 95% confidence interval 1.71 to 2.17), followed by internal comparators (1.52 (1.17 to 1.97) for self matching and 1.42 (1.01 to 1.99) for pregnancies before treatment), and ultimately women with disease but no treatment (1.27, 1.14 to 1.41). In women with untreated CIN, and in pregnancies before treatment, the risk of preterm birth compared with general population was significantly higher (1.24, 1.14 to 1.35). The subgroup analysis of the risk of preterm birth according to cone depth and comparison group showed a similar direction of effect, although for cone depth ≤10-12 mm the difference became insignificant. The number of studies was small for many comparisons. For treated versus untreated women with CIN there were four studies for cone depth ≤10-12 mm (43 145 women, 7.0% *v* 5.0%, relative risk 1.11, 95% confidence interval 0.85 to 1.43), four studies for cone depth ≥10-12 mm (45 275 women, 9.6% *v* 5.0%, 1.52, 1.37 to 1.68), three studies for cone depth ≥15-17 mm (33 934 women, 9.6% *v* 4.3%, 2.30, 1.57 to 3.35), and two studies for cone depth ≥20 mm (32 717 women, 9.3% *v* 4.2%, 4.32, 0.93 to 20.03) (table E, appendix 2). Furthermore, the sensitivity analysis that excluded studies that scored below the median Newcastle-Ottawa score (8.3) did not change the results of the analysis; similarly the results did not change when we excluded studies that scored ≤7 and ≤6 (data not shown). The subgroup analyses of studies based on the cohort selection or the comparability of the comparison groups showed similar direction and magnitude of effect (data not shown). The univariate meta-regression analysis suggested that the type of treatment and comparator significantly affected the risk of preterm birth, although the type of treatment and Newcastle-Ottawa score did not. These factors remained significant in a multivariate regression analysis. When we performed further meta-regression restricting only to excisional treatments and using as a comparator women with colposcopy/biopsy, we found that all treatments were associated with an increased risk of preterm birth (1.34, 1.10 to 1.64, for large loop excision of the transformation zone; 2.3, 1.39, 3.85, for cold knife conisation; 1.6, 0.91 to 2.87, for laser conisation; and 4.26, 1.96 to 9.33, for needle excision of the transformation zone).

**Table 4 tbl4:** Preterm birth (<37 weeks’ gestation) in women with cervical intraepithelial neoplasia (CIN) for treated and untreated women according to comparison group

Comparison	No of studies	Total No of women	No (%) of women		Effect estimate RR (95% CI)	P value for heterogeneity (I^2^%)
			Treated	Untreated		
**All treatment types *v* untreated external**						
Overall	46	5 193 761	5888/55 799 (10.6)	278 963/5 137 962 (5.4)	1.93 (1.71 to 2.17)	<0.001 (90)
CKC	7	37 370	62/390 (15.9)	2263/36 980 (6.1)	3.28 (2.44 to 4.42)	0.99 (0)
LC	6	1126	68/480 (14.2)	46/646 (7.1)	2.39 (1.24 to 4.61)	0.02 (63)
NETZ	1	7361	17/71 (23.9)	300/7290 (4.1)	5.82 (3.79 to 8.94)	N/E
LLETZ	20	1 415 006	1513/19 934 (7.6)	65 080/1 395 072 (4.7)	1.69 (1.46 to 1.97)	<0.001 (68)
LA	4	1258	37/510 (7.3)	50/748 (6.7)	1.27 (0.67 to 2.4)	0.19 (38)
CT	1	58	1/36 (2.8)	0/22 (0.0)	1.86 (0.08 to 43.87)	N/E
Excision NOS	12	3 101 232	3716/27 546 (13.5)	182 711/3 073 686 (5.9)	2.05 (1.61 to 2.60)	<0.001 (96)
Ablation NOS	5	588 949	430/6482 (6.6)	26 534/582 467 (4.6)	1.45 (1.26 to 1.67)	0.19 (35)
Treatment NOS	3	41 401	44/350 (12.6)	1979/41 051 (4.8)	2.20 (1.28 to 3.78)	0.07 (62)
**All treatment types *v* internal (pre-treatment pregnancies)**						
Overall	14	83 528	3117/22 121 (14.1)	3949/61 407 (6.4)	1.42 (1.01 to 1.99)	<0.001 (89)
CKC	3	1430	39/347 (11.2)	38/1083 (3.5)	1.79 (0.81 to 3.95)	0.15 (47)
LC	2	161	8/87 (9.2)	3/74 (4.1)	1.65 (0.11 to 23.58)	0.06 (7)
LLETZ	5	3331	192/1524 (12.6)	178/1807 (9.9)	1.21 (0.73 to 2.01)	0.002 (77)
LA	1	226	16/129 (12.4)	10/97 (10.3)	1.20 (0.57 to 2.53)	N/E
CT	1	180	3/115 (2.6)	2/65 (3.1)	0.85 (0.15 to 4.94)	N/E
Excision NOS	3	78 200	2859/19 919 (14.3)	3718/58 281 (6.4)	1.65 (0.88 to 3.08)	<0.001 (96)
**All treatment types *v* internal (self matching)**						
Overall	7	2916	157/1458 (10.8)	103/1458 (7.1)	1.52 (1.17 to 1.97)	0.36 (9)
LC	2	354	12/177 (6.8)	9/177 (5.1)	1.30 (0.56 to 3.06)	0.42 (0)
LLETZ	1	516	31/258 (12.0)	17/258 (6.6)	1.82 (1.04 to 3.21)	N/E
Excision NOS	3	1922	104/961 (10.8)	72/961 (7.5)	1.46 (0.89 to 2.39)	0.08 (60)
Treatment NOS	1	124	10/62 (16.1)	5/62 (8.1)	2.00 (0.73 to 5.51)	N/E
**All treatment types *v* untreated colposcopy+/−biopsy**						
Overall	13	74 958	2033/23 123 (8.8)	3119/51 835 (6.0)	1.27 (1.14 to 1.41)	<0.001 (55)
CKC	2	265	25/107 (23.4)	18/158 (11.4)	1.76 (1.01 to 3.08)	0.83 (0)
LC	1	177	20/105 (19.0)	9/72 (12.5)	1.52 (0.74 to 3.15)	N/E
LLETZ	9	39 249	877/10 441 (8.4)	1511/28 808 (5.2)	1.33 (1.11 to 1.6)	0.02 (55)
LA	2	3326	115/1228 (9.4)	182/2098 (8.7)	1.05 (0.84 to 1.31)	0.45 (0)
RD	1	2150	109/760 (14.3)	123/1390 (8.8)	1.62 (1.27 to 2.06)	N/E
Excision NOS	5	20 321	756/7933 (9.5)	961/12 388 (7.8)	1.23 (1.07 to 1.41)	0.2 (33)
Ablation NOS	2	9470	131/2549 (5.1)	315/6921 (4.6)	1.00 (0.74 to 1.36)	0.18 (45)
**All treatment types *v* untreated HSIL**						
Overall	3	3764	364/3022 (12.0)	58/742 (7.8)	1.37 (0.85 to 2.19)	0.05 (53)
CKC	1	103	7/67 (10.4)	1/36 (2.8)	3.76 (0.48 to 29.39)	N/E
NETZ	1	109	17/71 (23.9)	2/38 (5.3)	4.55 (1.11 to 18.66)	N/E
LLETZ	1	881	55/572 (9.6)	12/309 (3.9)	2.48 (1.35 to 4.55)	N/E
Excision NOS	2	2275	247/1955 (12.6)	38/319 (11.9)	1.06 (0.71 to 1.59)	0.24 (28)
Ablation NOS	2	397	38/357 (10.6)	5/40 (12.5)	0.68 (0.28 to 1.68)	0.87 (0)
**Untreated women *v* general population**						
Overall	17	4 359 362	6261/105 660 (5.9)	237 203/4 253 702 (5.6)	1.24 (1.14 to 1.35)	<0.001 (71)
Pregnancies before treatment	12	3 134 087	3893/60 543 (6.4)	176 453/3 073 544 (5.7)	1.26 (1.08 to 1.45)	0.03 (49)
Untreated colposcopy+/−biopsy	4	1 046 823	2310/44 375 (5.2)	49 646/1 002 448 (5.0)	1.22 (1.11 to 1.34)	0.01 (74)
Untreated HSIL	3	178 452	58/742 (7.8)	11104/177 710 (6.2)	1.40 (0.94 to 2.1)	0.08 (59)

CKC=cold knife conisation; CT=cryotherapy; HSIL=high grade squamous intraepithelial lesion; LA=laser ablation; LC=laser conisation; LLETZ=large loop excision of transformation zone; N/E=not eligible; NETZ=needle excision of transformation zone; NOS=not otherwise specified; RD=radical diathermy.

Several studies assessed other adverse maternal outcomes (table F, appendix 2), and many of these were found to be increased after cervical treatment.****This increase was more commonly associated with excisional than ablative techniques and with more radical treatment, although the number of studies assessing each individual treatment method was often small. Cervical treatment was associated with an increased risk of spontaneous overall, severe, and extreme preterm birth (<37 weeks: 14 studies, 1 024 731 women, 7.0% *v* 3.7%; relative risk 1.76, 95% confidence interval 1.47 to 2.11; <32-34 weeks: seven studies, 655 675 women, 1.8% *v* 0.6%; 2.63, 1.91 to 3.62; <28 weeks: two studies, 626 670 women, 0.6% *v* 0.2%, 3.18, 1.64 to 6.16) and admissions for threatened preterm birth (five studies, 903 women, 9.1% *v* 3.2%, 2.44, 1.37 to 4.33). The risk (<37 weeks) was higher for cold knife conisation (3.53, 2.05 to 6.05) followed by excision not otherwise specified (1.70, 1.17 to 2.46), large loop excision of the transformation zone (1.60, 1.22 to 2.08), and ablation not otherwise specified (1.42, 1.20 to 1.70). Needle excision of the transformation zone and laser ablation were each assessed in only one study. There was substantial heterogeneity for the comparisons assessing all gestational categories (P<0.05).

The risk of premature rupture of membranes (<37 weeks: 21 studies, 477 011 women; 6.1% *v* 3.4%, relative risk 2.36, 95% confidence interval 1.76 to 3.17) and chorioamnionitis (four studies, 29 198 women, 3.5 *v* 1.1%; 3.43, 1.36 to 8.64) was also increased after treatment. Risk was higher after cold knife conisation (4.11, 2.05 to 8.25) followed by large loop excision of the transformation zone (2.15, 1.48 to 3.12). Needle excision of the transformation zone was assessed in only one study, and laser ablation did not significantly affect the risk but was assessed in only two studies.

The mode of delivery (caesarean section or instrumental delivery), the length of labour (precipitous or prolonged), the use of analgesia (epidural, pethidine, or other), the rate of induction of labour (with or without oxytocin), cervical stenosis, and haemorrhage (antenatal or postpartum) were not affected by treatment. As expected, the rate of cervical cerclage insertion was higher for treated than non-treated women (eight studies, 141 300 women, 4.0% *v* 0.7%, relative risk 14.29, 95% confidence interval 2.85 to 71.65) and more so for cold knife conisation (31.42, 2.32 to 426.2), large loop excision of the transformation zone (11.0, 0.64 to 190), or excisional treatment not otherwise specified (42.45, 28.99 to 62.16).

### Neonatal outcomes

More than 30 studies assessed one or more neonatal outcomes (table G, appendix 2). Cervical treatment (excisional or ablative) was associated with a significant increase in adverse neonatal outcomes compared with outcomes in women who did not undergo treatment (comparison group not specified). The association with adverse neonatal events was stronger and more common for excisional rather than ablative techniques and with increasing treatment radicality, although the number of studies for each individual treatment technique was often limited.

More specifically, cervical treatment overall was associated with an increased risk of low birth weight (<2500 g: 30 studies, 1 348 206 women, 7.9% *v* 3.7%, relative risk 1.81, 95% confidence interval 1.58 to 2.07; <1500 g: five studies, 76 836 women, 2.0% *v* 0.5%, 3.00, 1.54 to 5.85), admission to a neonatal intensive unit (eight studies, 2557 women, 12.6% *v* 8.9%, 1.45, 1.16 to 1.81), and perinatal mortality (23 studies, 1 659 433 women, 0.9% *v* 0.7%, 1.51, 1.13 to 2.03). There was significant heterogeneity between studies for perinatal mortality (P=0.04, I^2^=36%).

The rate of neonates with birth weight <2500 g was significantly higher for women treated with cold knife conisation (five studies, 30 304, relative risk 2.51, 95% confidence interval 1.78 to 3.53), large loop excision of the transformation zone (12 studies, 3357, 2.11, 1.51 to 2.94), excisional (10 studies, 823 648, 2.01, 1.62 to 2.49) or ablative (four studies, 483 402, 1.36, 1.19 to 1.55) treatment not otherwise specified but not so for laser ablation (1.07, 0.59 to 1.92), although for that comparison there were only four studies with a total of 1104 participants. The rate of admission to neonatal intensive care was assessed only for excisional techniques and was significantly increased after large loop excision of the transformation zone (five studies, 1994 women, 1.42, 1.01 to 1.99). Perinatal mortality was significantly increased overall and for excisional technique not otherwise specified (five studies, 820 028, 1.85, 1.02 to 3.36) but not for the individual techniques, possibly because of the limited number of studies and the low prevalence of the outcome. Subgroup analysis according to the different comparison groups or cone depths was not possible because of the limited number of studies assessing each outcome.

## Discussion

### Main findings

The knowledge that local treatment for cervical precancer, particularly excisional, increases the risk of preterm birth has led to major changes in clinical practice. With a rapidly evolving evidence base and inconsistencies in the published literature,^14^ ^15^ ^17^ ^18^ ^66^ ^113^ a high quality synthesis of the evidence should be available for effective counselling of patients at colposcopy and antenatal clinics.

This meta-analysis shows that any local cervical treatment for preinvasive or early invasive disease increases the risk of preterm birth and adverse sequelae in a subsequent pregnancy, although the impact of small excisions, as opposed to just having the disease, remains uncertain and is likely to be small. Cervical treatment was found to be associated with an increased risk of overall, severe, and extreme prematurity, spontaneous preterm birth, threatened preterm labour, premature rupture of the membranes, chorioamnionitis, low birth weight, neonatal admission, and perinatal death. The rate of cervical cerclage was unsurprisingly substantially increased in treated women compared with untreated controls. Treatment equally affected outcomes for nulliparous as well as parous women and singleton and multiple pregnancies. The mode of delivery, length of labour, induction rate, use of analgesia, rate of stenosis, and haemorrhage were not significantly affected.

The magnitude of the effect of treatment was higher for more radical techniques (such as cold knife conisation followed by large loop excision of the transformation zone and laser ablation) and for excision rather than ablation. Multiple conisations increased the risk of preterm birth fourfold compared with untreated controls overall. Subgroup analyses clearly showed that the risk of preterm birth is directly correlated with the cone dimensions (depth/volume) and progressively increases with increasing cone depth (“dose effect”). Although the risk was increased even for excisions less than 10 mm in depth, this was almost twofold for excisions of more than 10 mm, threefold for more than 15-17 mm, and almost fivefold for excisions exceeding 20 mm in depth.

It has previously been suggested that the impact of treatment on the risk of preterm birth might not be a consequence of treatment but rather a product of other confounders present in women with cervical disease.^7^ ^14^ ^15^ Our subgroup analyses that stratified the risk by the comparator used, clearly documents that although the risk of preterm birth is significantly increased after treatment irrespective of the comparison group used, the choice of comparator might overinflate or underestimate the effect from treatment. The magnitude of effect was higher when we used external controls, followed by internal controls, followed by women who had disease but were not treated. The analyses in women with high grade squamous intraepithelial lesion but no treatment only included three studies and 3764 participants; we were unable to draw any firm conclusions from this comparison. When we assessed the risk of preterm birth according to both the cone depth and comparator, we noted overall the same direction of effect. Although the difference in the risk of preterm birth for small excisions (**≤**10-12 mm) compared with just having CIN but no treatment, became insignificant, the number of studies assessing that comparison was small, and we cannot draw firm conclusions.

Our results also confirm that although women with CIN have a higher baseline risk of prematurity than the general population, cervical treatment, and particularly deep cones, further increase that risk.

### Strengths and limitations

This is the first systematic review to show that any local cervical treatment technique (excisional or destructive) is associated with an increased risk of preterm birth and adverse obstetric sequelae and to document that the risk directly correlates to the cone depth (and volume), the treatment technique (excision more than ablation), and radicality. This meta-analysis included a large number of studies (71 cohorts) with sufficient sample size and power to explore several comparisons of treatment techniques and cone depths. Furthermore, we were able to perform subgroup analyses according to the comparator used and quantify the risk in different clinical groups.

The results, however, should be interpreted with caution. Because of the premalignant nature of the disease, no randomised studies could be identified. All the included studies were cohorts, nearly all retrospective. Such reports are at known risk of recall bias and inadequate adjustment for known and unknown confounders, while some of the outcomes of interest were difficult to measure objectively. Many of the studies relied on data collected from structured interviews and mailed questionnaires, and in some of these the response rate was small, also increasing the risk of incomplete outcome data (attrition) and misclassification bias. The studies often had different designs and used comparisons between and among women and mixed matching. Although the overall number of studies was large, for some outcomes and comparisons there were few studies, and the analyses did not have sufficient sample sizes to support definite conclusions.

Although heterogeneity between studies was not significant for most of the analyses, some subgroup analyses did show variation in the outcomes across studies. This was often in analyses that included small number of studies and participants. Meta-regression was possible for some but not all possible confounders. For many moderators, data were reported only in a proportion of the included studies. When these studies were not deemed representative of the whole population of studies, we did not perform meta-regression as this would introduce bias. Sensitivity and subgroups analyses based on study quality did not change the effect of the meta-analysis.

There were further limitations in the interpretation of the data. The gestational age cut offs used for the definitions of severe and extreme prematurity and for different cone depths varied slightly across studies; we merged these in broader groups for the analysis. Individual patient meta-analysis data are required to more accurately describe the stratified risk of preterm birth for individual cone depths. The data on cone dimensions relied on retrospective data recorded in histopathology reports of formalin fixed samples, with obvious limitations. The formulas used for the calculation of volume also varied across studies. Future research should aim to correlate outcomes with precise prospective cone depth and cervical measurements.

Both the included and excluded studies showed a wide range of inclusion/exclusion criteria and outcome measures limiting statistical pooling of all the primary studies. There should be agreement among colposcopists and obstetricians on core research clinical outcome measures in line with the CROWN initiative of the premier reproductive health journals.^134^ This would improve the applicability of findings of primary and secondary research internationally.

### Interpretation in light of other evidence

With an increasing evidence base suggesting that this risk is higher for more radical techniques, there has been a tendency to use less aggressive treatments.^5^ Although it was previously thought that the various techniques had comparable efficacy,^135^ evidence from a population based study raised concerns that less radical treatment could increase the risk of invasion after treatment.^136^ ^137^ Although the decreased number of hysterectomies could explain this increase, the move to less radical local conservative treatments is another plausible explanation. Additionally, since the first documentation of the reproductive risk associated with treatment almost a decade ago,^1^ subsequent observational studies and even meta-analyses reached contradictory conclusions^2^ ^3^ ^4^ ^16^ ^17^ ^18^ ^19^ and initiated debates within the scientific community. With some authors raising concerns that the progressive reduction in the radicality of treatment has led to increased risk of future of invasion,^136^ ^137^ and others advocating the move to less radical techniques like laser ablation for the prevention of future perinatal morbidity and mortality associated with treatment,^138^ high quality synthesis of the evidence had become an urgent unmet need. Some of the previous small meta-analyses had methodological flaws and attempted analysis of individual treatment techniques or subgroups, thereby minimising the validity of their findings in context with the rest of the literature.^16^ ^17^ ^18^ All the published meta-analyses failed to analyse the data according to major confounders and stratifiers of risk, the comparison group, and the depth of the excision. Although Bruinsma and Quinn first approached the comparison group as a possible confounder, data on the depth and dimensions of the treatment were not available.^4^

Preterm birth is a major cause of neonatal death and disability and represents an enormous cost to health services and society. While pregnant, these women with a history of cervical treatment make up a large proportion of referrals to specialist preterm labour prevention clinics. These referrals have increased from almost none in 1999, to more than 40% in 2012.^139^ Ultrasound directed surveillance is labour intensive, costly, and can be associated with maternal anxiety, more so because 85% of women after excision are effectively low risk and will deliver at term.^1^ ^4^

With rapidly accumulating evidence correlating cervical treatment to adverse reproductive morbidity, quantification of the comparative obstetric morbidity for different treatment techniques and cone depths is required to assist clinicians’ decision making and counselling. The results of our meta-analysis will allow clinicians, patients, and policy makers to balance the absolute increase in reproductive morbidity with increasing treatment radicality. Patients should be informed that treatment increases the risk of preterm birth compared with having CIN only, but the absolute increase in risk in small type 1 excisions is likely to be low, if any.

Furthermore, the quantified individual risk stratified by treatment and cone depth could allow obstetricians to select women considered to be at high risk of preterm birth who would benefit from intensive surveillance antenatally and minimise the unnecessary interventions for those at low risk. The antenatal management of women after treatment has been inconsistent and largely unit or clinician dependent.^30^ The risks and benefits associated with various interventions in pregnant women with a history of cervical treatment have not been fully assessed in properly designed studies.^140^ Future research should assess their value in this distinct clinical group and devise a logical prevention strategy.

### Conclusion

Women with CIN have a higher baseline risk of preterm birth than women from the general population. Local cervical treatment for preinvasive or early invasive disease further increases the risk, more so for excisional but also for ablative techniques. The risk of preterm birth increases with increasing cone depth (and volume) and techniques that remove or destroy larger parts of the cervix. The increase in risk for small excisions compared with having CIN is likely to be small, if any; more data are required.

In the decision to treat women of reproductive age, every effort should be made to perform a local treatment that will optimise the chances of a healthy pregnancy without compromising the completeness of the local treatment. Quality assurance in treatment of disease should include audit of dimensions of excisional specimens and persistent disease rates to ensure that treatment depth is kept to acceptable parameters (that is, at least 8 mm to include the crypts) and that oncological outcomes are not compromised.

Future research should investigate whether women who have preinvasive cervical disease are susceptible to both the disease and preterm birth, or whether HPV induced disease alone is the principal factor in increasing premature delivery. It is likely that a combination of immunological and other factors play a role. The uptake of prophylactic vaccination has been mixed in the developed world and minimal in low income countries. The impact of cervical treatment will continue to be relevant for many decades, and therefore robust clinical research in this field should remain a priority.

**What is already known on this topic**

Local cervical treatment has been associated with an increased risk of preterm birth, perinatal morbidity, and mortality in a subsequent pregnancy, which could be associated with depth of excision.
Discrepancies exist regarding the impact of treatment on the risk of subsequent preterm birth and whether CIN acts as a confounder, which might be caused by heterogeneity in comparison groups used in previous studies or different excision depths and/or treatment techniques that have been analysed

**What this study adds**

Increased risk of adverse obstetric outcomes is associated with the treatment technique (excision more than ablation) and radicality, determined by the depth and dimensions of the cone
Although the risk of preterm birth is higher after local treatment for CIN irrespective of the cone depth, the risk increases with increasing cone depth. The increase in risk in small excisions compared with just having CIN remains uncertain and is likely to be small, if any; more data are required
Choice of comparison group might overinflate or underestimate the effect from treatment because of the background increased risk of preterm birth in women with CIN. The increased risk of preterm birth, however, remains significantly increased after treatment, despite the chosen comparator and even in comparisons with women with CIN but no treatment

## Web Extra

Extra material supplied by the author

**Appendix 1:** Search strategy

**Appendix 2:** Supplementary tables A-F

**Appendix 3:** Forest plot for all treatment techniques

## Notes

Ethical approval: Not required.

Data sharing: No additional data available.

Transparency: The lead author affirms that the manuscript is an honest, accurate, and transparent account of the study being reported; that no important aspects of the study have been omitted; and that any discrepancies from the study as planned have been explained.
