# Supplementary material for: Adverse obstetric outcomes after local treatment for cervical preinvasive and early invasive disease according to cone depth: systematic review and meta-analysis
Source: BMJ. 2016 Jul 28;354:i3633. doi: 10.1136/bmj.i3633 (PMC4964801; doi:10.1136/bmj.i3633)
Supplement: Supplementary file 1 — Appendix 1: Search strategy [file kyrm031600.ww1_default.pdf]

Appendix 1: Search strategy [posted as supplied by author]

Medline Ovid

1 exp Uterine Cervical Neoplasms/
2 (cervi* and (cancer* or tumor* or tumour* or neoplas* or malignan* or carcinom*)).mp.
3 exp Cervical Intraepithelial Neoplasia/
4 CIN.mp.
5 (cervi* and (intraepithel* or epithel* or dysplasia or pre-cancer* or precancer*)).mp.
6 or/1-5
7 exp Conization/
8 (conisation or conization).mp.
9 exp Laser Therapy/
10 laser.mp.
11 exp Cryotherapy/
12 cryotherapy.mp.
13 cold coagulation.mp.
14 exp Diathermy/
15 diatherm*.mp.
16 cone biopsy.mp.
17 loop.mp.
18 LLETZ.mp.
19 LEEP.mp.
20 ablat*.mp.
21 excision*.mp.
22 transformation zone.mp.
23 (CKC or LA or LC or CC or RD or TZ).mp.
24 (conservative and (method* or treatment* or intervention* or management)).mp.
25 or/7-24
26 6 and 25
27 exp Premature Birth/
28 (preterm or premature).mp.
29 exp Infant, Low Birth Weight/
30 birth weight.mp.
31 Perinatal Mortality/
32 perinatal mortality.mp.
33 exp Intensive Care, Neonatal/
34 (neonatal and intensive care).mp.
35 exp Fertility/
36 fertil*.mp.
37 conception.mp.
38 exp Pregnancy/
39 pregnancy.mp.
40 gestation*.mp.
41 exp Abortion, Spontaneous/
42 miscarriage*.mp.
43 exp Cesarean Section/
44 (cesarean or caesarean).mp.
45 exp Obstetric Labor, Premature/
46 exp Labor, Obstetric/
47 (labor or labour).mp.
48 Fetal Membranes, Premature Rupture/
49 pPROM.mp.
50 or/27-49
51 26 and 50
key:
mp=title, original title, abstract, name of substance word, subject heading word

Embase Ovid

1 exp uterine cervix tumor/
2 (cervi* and (cancer* or tumor* or tumour* or neoplas* or malignan* or carcinom*)).mp.
3 uterine cervix carcinoma in situ/
4 CIN.mp.
5 (cervi* and (intraepithel* or epithel* or dysplasia or pre-cancer* or precancer*)).mp.
6 or/1-5
7 uterine cervix conization/
8 (conisation or conization).mp.
9 low level laser therapy/
10 laser.mp.
11 exp cryotherapy/
12 cryotherapy.mp.
13 cold coagulation.mp.
14 diathermy/
15 diatherm*.mp.
16 cone biopsy.mp.
17 loop.mp.
18 LLETZ.mp.
19 LEEP.mp.
20 ablat*.mp.
21 excision*.mp.
22 transformation zone.mp.
23 (CKC or LA or LC or CC or RD or TZ).mp.
24 (conservative and (method* or treatment* or intervention* or management)).mp.
25 or/7-24
26 6 and 25
27 prematurity/
28 (preterm or premature).mp.
29 exp low birth weight/
30 birth weight.mp.
31 perinatal mortality/
32 perinatal mortality.mp.
33 newborn intensive care/
34 (neonat* and intensive care).mp.
35 female fertility/
36 fertil*.mp.
37 conception/
38 conception.mp.
39 exp pregnancy/
40 pregnancy.mp.
41 gestation*.mp.
42 spontaneous abortion/
43 miscarriage*.mp.
44 cesarean section/
45 (cesarean or caesarean).mp.
46 premature labor/
47 (labor or labour).mp.
48 premature fetus membrane rupture/
49 pPROM.mp.
50 or/27-49
51 26 and 50

key:
mp=title, abstract, subject headings, heading word, drug trade name, original title, device manufacturer, drug manufacturer name

CENTRAL

#1 MeSH descriptor **Uterine Cervical Neoplasms** explode all trees
#2 cervi* and (cancer* or tumor* or tumour* or neoplas* or malignan* or carcinom*)
#3 MeSH descriptor **Cervical Intraepithelial Neoplasia** explode all trees
#4 CIN
#5 cervi* and (intraepithel* or epithel* or dysplasia or pre-cancer* or precancer*)
#6 (#1 OR #2 OR #3 OR #4 OR #5)
#7 MeSH descriptor **Conization** explode all trees
#8 conisation or conization
#9 MeSH descriptor **Laser Therapy** explode all trees
#10 laser
#11 MeSH descriptor **Cryotherapy** explode all trees
#12 cryotherapy
#13 cold coagulation
#14 MeSH descriptor **Diathermy** explode all trees
#15 diatherm*
#16 cone biopsy
#17 loop
#18 LLETZ
#19 LEEP
#20 ablat*
#21 excision*
#22 transformation zone
#23 CKC or LA or LC or CC or RD or TZ
#24 conservative and (method* or treatment* or intervention* or management)
#25 (#7 OR #8 OR #9 OR #10 OR #11 OR #12 OR #13 OR #14 OR #15 OR #16 OR #17 OR #18 OR #19 OR #20 OR #21 OR #22 OR #23 OR #24)
#26 (#6 AND #25)
#27 MeSH descriptor **Premature Birth** explode all trees
#28 preterm or premature
#29 MeSH descriptor **Infant, Low Birth Weight** explode all trees
#30 birth weight
#31 MeSH descriptor **Perinatal Mortality** explode all trees
#32 perinatal mortality
#33 MeSH descriptor **Intensive Care, Neonatal** explode all trees
#34 neonat* and (intensive care)
#35 MeSH descriptor **Fertility** explode all trees
#36 fertil*
#37 conception
#38 MeSH descriptor **Pregnancy** explode all trees
#39 pregnancy
#40 gestation*
#41 MeSH descriptor **Abortion, Spontaneous** explode all trees
#42 miscarriage*
#43 MeSH descriptor **Cesarean Section** explode all trees
#44 cesarean or caesarean
#45 MeSH descriptor **Obstetric Labor, Premature** explode all trees
#46 MeSH descriptor **Labor, Obstetric** explode all trees
#47 labor or labour
#48 MeSH descriptor **Fetal Membranes, Premature Rupture** explode all trees
#49 pPROM
#50 (#27 OR #28 OR #29 OR #30 OR #31 OR #32 OR #33 OR #34 OR #35 OR #36 OR #37 OR #38 OR #39 OR #40 OR #41 OR #42 OR #43 OR #44 OR #45 OR #46 OR #47 OR #48 OR #49)
#51 (#26 AND #50)
