# Supplementary material for: Adverse obstetric outcomes after local treatment for cervical preinvasive and early invasive disease according to cone depth: systematic review and meta-analysis
Source: BMJ. 2016 Jul 28;354:i3633. doi: 10.1136/bmj.i3633 (PMC4964801; doi:10.1136/bmj.i3633)
Supplement: Supplementary file 2 — Appendix 2: Supplementary tables A-F [file kyrm031600.ww2_default.pdf]

Appendix 2: Supplementary tables [posted as supplied by author]

Table A: Characteristics of included studies assessing obstetric outcomes for treated versus untreated women.

Study (Country)	Study Design	Comparison Group	Procedure	Treated*	Untreated*	Source of data	Outcomes	Newcastle-Ottawa score
Jones 1979 (UK)	Retrospective cohort (population-based)	External: matching for age, parity, social class, delivery date, singleton birth	CKC	66	264	Clinical records from Cardiff Cervical Cytology Study - Cardiff Birth Survey (registry)	PTB (<37w); PTB (<37w)(singleton); sPTB (<37w); CS; ID; PrecL (<2h); ProlL (>12h); LBW (<2500g); PM; SB	9
Weber 1979 (Denmark)	Retrospective cohort (hospital-based)	External: matching for age	CKC	48	48	Hospital records; structured interviews	LBW (<2500g)	8
Buller 1982 (USA)	Retrospective cohort (hospital-based)	Internal (pre-treatment pregnancies)	CKC	47	79	Hospital records	PTB (<37w); tPTL; CS	7
Hemmingsson 1982 (Sweden)	Retrospective cohort (hospital-based)	Internal (pre-treatment pregnancies)	CT	115	65	Hospital records	PTB (<36w); pPROM; CS; stenosis; PM	8
Larsson 1982 (Sweden)	Retrospective cohort (population-based)	Internal (pre-treatment pregnancies) matching for age, parity, socioeconomic status, smoking, treatment, diseases	CKC	197	284	South Swedish Regional Tumour Registry, hospital records	PTB (<37w); PTB (<37w)(singleton); PTB (<37w)(multiple); PM; SB	9
Ludviksson 1982 (Sweden)	Retrospective cohort (hospital-based)	External: matching for age, parity, time of delivery	CKC	83	79	Hospital records	PTB (≤37w); PTB (≤33w); PTB (<30w); PPH; MOH	8
Moinian 1982 (Sweden)	Retrospective cohort (hospital-based)	Internal (pre-treatment pregnancies)	CKC	103	720	Hospital records	PTB (<37w); cerclage	8

Study (Country)	Study Design	Comparison Group	Procedure	Treated*	Untreated*	Source of data	Outcomes	Newcastle-Ottawa score
Jones 1979 (UK)	Retrospective cohort (population-based)	External: matching for age, parity, social class, delivery date, singleton birth	CKC	66	264	Clinical records from Cardiff Cervical Cytology Study - Cardiff Birth Survey (registry)	PTB (<37w); PTB (<37w)(singleton); sPTB (<37w); CS; ID; PrecL (<2h); ProlL (>12h); LBW (<2500g); PM; SB	9
Anderson 1984 (UK)	Retrospective cohort (hospital-based)	External: matching for age, race, births, miscarriages/TOPs	LA	68	70	Hospital records; postal questionnaires; obstetricians	PTB (<37w); PTB (<37w)(single); CS; ID; ProlL (>12h); LBW (<2500g)	7
Kristensen 1985 (Denmark)	Retrospective cohort (population-based)	External: matching for age, parity	Treatment NOS	85	12792	Hospital records; questionnaires	PTB (<37w); PTB (<37w)(singleton); LBW (<2500g)	9
Kuoppala 1986 (Finland)	Retrospective cohort (hospital-based)	External: matching for age, parity, date of delivery, singleton birth	CKC	62	62	Hospital records	PTB (<37w); CS; ID; IoL; oxytocin; analgesia; cerclage; PM; SB	9
Saunders 1986 (UK)	Retrospective cohort (hospital-based)	External: matching for age, parity, race, year of delivery, singleton pregnancy	LA	97	97	Hospital records; general practitioners	PTB (<37w); PTB (<37w)(single); PTB (<37w)(repeat); pPROM; CS; ID; LBW (<2500g); PM	6
Gunasekera 1992 (UK)	Retrospective cohort (hospital-based)	External: matching for age, parity, race, duration of pregnancy, smoking	LLETZ; LA	140 (LLLETZ=23; LA=117)	140 (LLLETZ=23; LA=117)	Hospital records	PTB (<37w); CS; ID; ProlL(>12h)	9
Blomfield 1993 (UK)	Retrospective cohort (hospital-based)	External: matching for age, parity, ethnic group	LLETZ	40	80	Hospital records	PTB (<37w); sPTB (<37w); CS; ID; IoL; oxytocin; epidural; LBW (<2500g); NICU; PM	9
Haffenden 1993 (UK)	Retrospective cohort (hospital-based)	External: matching for age, parity	LLETZ	152	152	Hospital records	PTB (<37w); CS; ID; PrecL (<2h); ProlL (>12h); IoL; oxytocin; epidural; LBW (<2500g)	9

Study (Country)	Study Design	Comparison Group	Procedure	Treated*	Untreated*	Source of data	Outcomes	Newcastle-Ottawa score
Jones 1979 (UK)	Retrospective cohort (population-based)	External: matching for age, parity, social class, delivery date, singleton birth	CKC	66	264	Clinical records from Cardiff Cervical Cytology Study - Cardiff Birth Survey (registry)	PTB (<37w); PTB (<37w)(singleton); sPTB (<37w); CS; ID; PrecL (<2h); ProlL (>12h); LBW (<2500g); PM; SB	9
Hagen 1993 (Norway)	Retrospective cohort (hospital-based)	External: matching for age, parity; regression for height, marital status, education, smoking, TOP - index pregnancy: hypertension, APH, mode of delivery	LC	56	112	Hospital records	PTB (≤37w); PTB (≤37w)(nulliparous); PTB (≤37w)(parous); PTB (≤37w)(singleton); CS; ID; APH	9
Kristensen 1993 (Denmark)	Retrospective cohort (population-based)	A) External: no matching, no regression B) Internal (self-matching)	Treatment NOS (CKC, laser, electrocaute-ry)	A) 130<br>B) 62	A) 28124<br>B) 62	Medical Birth Register; national Register of Hospital Discharges	PTB (<37w); PTB (<37w)(nulliparous); PTB (<37w)(parous); PTB (<37w)(singleton)	7
Braet 1994 (UK)	Retrospective cohort (hospital-based)	External: matching for age, parity, smoking	LLETZ	78	78	Hospital records	PTB (<37w); PTB (<37w)(singleton); pPROM; CS; ID; APH; LBW (<2500g); PM	9
Cruickshank 1995 (UK)	Retrospective cohort (hospital-based)	A) External: age, parity, partner's social class, height, smoking<br>B) Internal (pre-treatment pregnancies)	LLETZ	149	A) 298<br>B) 133	Aberdeen Maternity and Neonatal Databank; postal questionnaires	PTB (<37w); PTB (<28w); PTB (singleton)(<37w); CS; PrecL (<2h); SB	7
Sagot 1995 (France)	Retrospective cohort (hospital-based)	Internal (pre-treatment pregnancies)	LC	53	59	Hospital records	PTB (<37w); tPTL; pPROM; CS; chorioamnionitis; cerclage	7

Study (Country)	Study Design	Comparison Group	Procedure	Treated*	Untreated*	Source of data	Outcomes	Newcastle-Ottawa score
Jones 1979 (UK)	Retrospective cohort (population-based)	External: matching for age, parity, social class, delivery date, singleton birth	CKC	66	264	Clinical records from Cardiff Cervical Cytology Study - Cardiff Birth Survey (registry)	PTB (<37w); PTB (<37w)(singleton); sPTB (<37w); CS; ID; PrecL (<2h); ProlL (>12h); LBW (<2500g); PM; SB	9
Spitzer 1995 (Jamaica)	Retrospective cohort (hospital-based)	Internal (pre-treatment pregnancies) with matching for age, parity	LC; LA	163 (LC=34; LA=129)	112	Hospital/private practice records; questionnaires (by mail, phone or in person)	PTB (<37w)	7
Bekassy 1996 (Sweden)	Retrospective cohort (hospital-based)	A) External: matching for age, parity, time of delivery<br>B) Internal (self-matching)	LC ('miniconisa-tion')	A) 250<br>B) 148	A) 250<br>B) 148	National Medical Birth Registry; hospital records	PTB (<37w); PTB (<37w)(nulliparous); PTB (<37w)(parous); PTB (<37w)(single); PTB (<37w)(repeat); CS; ID; ProlL (>12h); stenosis; LBW (<2500g); PM; SB	8
Forsmo 1996 (Norway)	Retrospective cohort (hospital-based)	External: age, parity, place of delivery	LC; LA	71 (LC=51; LA=20)	174	Hospital records, postal questionnaires	LBW (<2500g); LBW (<2000g); LBW (<1500g); PM; SB	8
Turlington 1996 (USA)	Retrospective cohort (hospital-based)	Biopsy but no treatment: regression for age	LLETZ	15	15	Hospital records; telephone interviews/mail-in questionnaires	SB	7
Raio 1997 (Switzerland)	Retrospective cohort (hospital-based)	A) External: matching for age, parity, marital status, social class, smoking, PTB<br>B) Internal (self-matching)	LC	A) 64<br>B) 26	A) 64<br>B) 26	Hospital records	PTB (<37w); PTB (<37w)(singleton); PTB (<37w)(D<10mm); PTB (<37w)(D≥10mm); pPROM	9
Andersen 1999 (Denmark)	Retrospective cohort (hospital-based)	External: matching for age, parity	LC	75	150	Hospital records	PTB (≤37w); PTB (≤37w)(D<15mm); PTB (≤37w)(D=15-20mm); PTB (≤37w)(D>20mm); pPROM; CS; PM; SB	9

Study (Country)	Study Design	Comparison Group	Procedure	Treated*	Untreated*	Source of data	Outcomes	Newcastle-Ottawa score
Jones 1979 (UK)	Retrospective cohort (population-based)	External: matching for age, parity, social class, delivery date, singleton birth	CKC	66	264	Clinical records from Cardiff Cervical Cytology Study - Cardiff Birth Survey (registry)	PTB (<37w); PTB (<37w)(singleton); sPTB (<37w); CS; ID; PrecL (<2h); ProlL (>12h); LBW (<2500g); PM; SB	9
El-Bastawissi 1999 (USA)	Retrospective cohort (population-based)	A) External: matching for age, country<br>B) HSIL but no treatment<br>Both regression for parity, race, smoking, marital status, TOPs	Excision NOS (CKC, LC, LLETZ); Ablation NOS (LA, CT)	1096	A) 9201<br>B) 330	Cancer Surveillance System (a population-based cancer registry); Birth Certificates (from the Department of Health in Washington state)	PTB (<37w); PTB (<37w)(singleton); CS; LBW (<2500g)	9
van Rooijen 1999 (Sweden)	Retrospective cohort (hospital-based)	External: matching for age, parity, year of delivery	LA	236	472	Hospital records	PTB (<37w); PTB (<37w)(single); CS; APH; LBW (<2500g); LBW (<2000g); LBW (<1500g); LBW (<1000g)	9
Paraskevaidis 2002 (Greece)	Retrospective cohort (hospital-based)	External: matching for age, parity, smoking, multiple pregnancies, PTBs	LLETZ (for microinva-sion)	28≥	28	Hospital records	PTB (<37w); PTB (<37w)(single); PTB (<37w)(repeat); sPTB; CS; PrecL (<2h); LBW (<2500g); NICU	9
Sadler 2004 (New Zealand)	Retrospective cohort (hospital-based)	Colposcopy but no treatment: regression for age, ethnicity, socioeconomic status, smoking, obstetric history, transfer to hospital, APH	LC; LLETZ; LA	652	426	Hospital records	PTB (<37w); PTB (<37w)(single); PTB (<37w)(repeat); PTB (<37w)(singleton); PTB (<37w)(D≤10mm); PTB (<37w)(D=11-16mm); PTB (<37w)(D≥17mm); PTB (<32w); sPTB (<37w); pPROM	9
Tan 2004 (UK)	Retrospective cohort (hospital-based)	External: matching for age, parity	LLETZ	119	119	Hospital records	PTB (<37w); CS; ID; ProlL (>12h); IoL; oxytocin; epidural; pethidine	8

Study (Country)	Study Design	Comparison Group	Procedure	Treated*	Untreated*	Source of data	Outcomes	Newcastle-Ottawa score
Jones 1979 (UK)	Retrospective cohort (population-based)	External: matching for age, parity, social class, delivery date, singleton birth	CKC	66	264	Clinical records from Cardiff Cervical Cytology Study - Cardiff Birth Survey (registry)	PTB (<37w); PTB (<37w)(singleton); sPTB (<37w); CS; ID; PrecL (<2h); ProlL (>12h); LBW (<2500g); PM; SB	9
Acharya 2005 (Norway)	Retrospective cohort (hospital-based)	A) External: matching for age, parity, date of delivery, smoking, obstetric history<br>B) Internal (pre-treatment pregnancies)	LLETZ	79	A) 158<br>B) 45	Hospital records	PTB (<37w); tPTL; chorioamnionitis; IoL; LBW (<2500g); PM	9
Samson 2005 (Canada)	Retrospective cohort (hospital-based)	External: matching for age, parity, smoking status, year of delivery	LLETZ	571	571	Registries	PTB (<37w); PTB (<37w)(single); PTB (<37w)(repeat); PTB (<37w)(singleton); PTB (<37w)(multiple); PTB (<34w); PTB (<34w)(multiple); pPROM; CS; IoL; oxytocin; LBW (<2500g); NICU; PM; SB	9
Crane 2006 (Canada)	Retrospective cohort (hospital-based)	External: regression for age, gestation at USS, parity, smoking, APH, sPTB	CKC; LLETZ; CT	132 (CKC=21; LLETZ=75; CT=36)	81	Hospital records	sPTB (<37w); sPTB (<37w)(singleton); sPTB (<34w); CS; IoL; APH; LBW (<2500g); NICU; PM; Apgar (<7)(5min)	8
Klaritsch 2006 (Austria)	Retrospective cohort (hospital-based)	External: no matching, no regression	CKC	76	29711	Hospital records	PTB(<37w); PTB (<37w)(single); PTB (<37w)(singleton); PTB(<34w); pPROM; CS; chorioamnionitis; LBW (<2500g); PM	7

Study (Country)	Study Design	Comparison Group	Procedure	Treated*	Untreated*	Source of data	Outcomes	Newcastle-Ottawa score
Jones 1979 (UK)	Retrospective cohort (population-based)	External: matching for age, parity, social class, delivery date, singleton birth	CKC	66	264	Clinical records from Cardiff Cervical Cytology Study - Cardiff Birth Survey (registry)	PTB (<37w); PTB (<37w)(singleton); sPTB (<37w); CS; ID; PrecL (<2h); ProlL (>12h); LBW (<2500g); PM; SB	9
Bruinsma 2007 (Australia)	Retrospective cohort (hospital-based)	A) Colposcopy before pregnancy but no treatment B) Colposcopy during pregnancy but no treatment Both regression for age, drug use, marital status, medical conditions, TOPs, miscarriages, PTBs, treatment	CKC; LLETZ; LA; RD	1951	A) 2294<br>B) 1303	Hospital records and registries	PTB (<37w); PTB (<37w)(singleton); PTB (<32w); PTB (<28w); sPTB; pPROM; CS; ID; LBW (<2500g); PM; SB	9
Himes 2007 (USA)	Retrospective cohort (hospital-based)	Biopsy but no treatment – no matching, regression	LLETZ	114	962	Hospital records	PTB (<37w); PTB (<37w)(singleton); sPTB; pPROM	8
Jakobsson 2007 (Finland)	Retrospective cohort (population-based)	External: regression for age, parity, smoking	Excision NOS (CKC, LC, LLETZ); Ablation NOS (LA, CT, electrocoa-gulation)	8422 (Excision NOS=4846; Ablation NOS=3576)	1056855	National registers	PTB (<37w); PTB (<28w); LBW (<2500g); PM	9
Sjoborg 2007 (Norway)	Retrospective cohort (population-based)	A) External: matching for age, parity, plurality B) Internal (self-matching) Both regression for smoking, marital status, education	Excision NOS (LC, LLETZ)	A) 742 (LC=609; LLETZ=133)<br>B) 419	A) 742<br>B) 419	Hospital records	PTB (<37w); PTB (<32w); PTB (<28w); pPROM; LBW (<2500g); LBW (<1500g); LBW (<1000g); PM	8

Study (Country)	Study Design	Comparison Group	Procedure	Treated*	Untreated*	Source of data	Outcomes	Newcastle-Ottawa score
Jones 1979 (UK)	Retrospective cohort (population-based)	External: matching for age, parity, social class, delivery date, singleton birth	CKC	66	264	Clinical records from Cardiff Cervical Cytology Study - Cardiff Birth Survey (registry)	PTB (<37w); PTB (<37w)(singleton); sPTB (<37w); CS; ID; PrecL (<2h); ProlL (>12h); LBW (<2500g); PM; SB	9
Albrechtsen 2008 (Norway)	Retrospective cohort (population-based)	A) External<br>B) Internal (pre-treatment pregnancies)<br>Both regression for age, birth order	Excision NOS (CKC, LC, LLETZ)	14882	A) 2155505<br>B) 56927	National registries	PTB (<37w); PTB (<33w); PTB (<28w)	9
Parikh 2008 (USA)	Retrospective cohort (hospital-based)	External: no matching, no regression	LLETZ	87	18042	Hospital records	PTB (≤34w)	6
Jakobsson 2009 (Finland)	Retrospective cohort (hospital-based)	A) External: no matching<br>B) Internal (self-matching)<br>Both regression for age, parity, or both	LLETZ	A) 624<br>B) 258	A) 554507<br>B) 258	National registers and hospital records	PTB (<37w)(nulliparous); PTB (<37w)(parous)	8
Noehr 2009 (singletons & cone depth) (Denmark)	Retrospective cohort (population-based)	A) External<br>B) Biopsy but no treatment<br>Both regression for age, year of delivery, smoking, marital status	LLETZ; Ablation NOS	10207 (LLETZ=8180; Ablation NOS=2027)	A) 510841<br>B) 31630	National registries	sPTB (<37w); sPTB (<37w)(D≤12mm); sPTB (<37w)(D=13-15mm); sPTB (<37w)(D=16-19mm); sPTB (<37w)(D≥20mm); sPTB (<37w)(single); sPTB (<37w)(repeat); sPTB (<37w)(singleton); sPTB (<32w); sPTB (<28w)	9
Noehr 2009 (twins) (Denmark)	Retrospective cohort (population-based)	External: regression for age, year of delivery, smoking, marital status, IVF	LLETZ	166	9702	National registries	sPTB (<37w)(multiple); sPTB (<32w)(multiple); sPTB (<28w)(multiple)	9

Study (Country)	Study Design	Comparison Group	Procedure	Treated*	Untreated*	Source of data	Outcomes	Newcastle-Ottawa score
Jones 1979 (UK)	Retrospective cohort (population-based)	External: matching for age, parity, social class, delivery date, singleton birth	CKC	66	264	Clinical records from Cardiff Cervical Cytology Study - Cardiff Birth Survey (registry)	PTB (<37w); PTB (<37w)(singleton); sPTB (<37w); CS; ID; PrecL (<2h); ProlL (>12h); LBW (<2500g); PM; SB	9
Shanbhag 2009 (UK)	Retrospective cohort (population-based)	A) External<br>B) CIN3 but no treatment<br>Both regression for age, smoking, socioeconomic status, year of delivery, birth weight, malpresentation, sPTB, pPROM	Excision NOS (CKC, LC, LLETZ); Ablation NOS (LA, CC, diathermy coagulation)	1388 (Excision NOS=1103; Ablation NOS=285)	A) 119216<br>B) 87	National registries	PTB (<37w); sPTB (<37w); pPROM; CS; LBW (<2500g); PM	8
Fischer 2010 (USA)	Prospective cohort study (hospital-based)	External: matching for age, race, vaginal deliveries, gestational age at USS	Excision NOS (CKC, LLETZ)	85 (CKC=48; LLETZ=68; both=2)	85	Hospital records	PTB (<37w); PTB (<37w)(singleton); PTB (<34w); CS; cerclage	8
Ortoft 2010 (Denmark)	Retrospective cohort (hospital-based)	A) External B) HSIL but no treatment<br>Both regression for age, parity, smoking, education, marital status<br>C) Internal (self-matching)	CKC; NETZ; LLETZ	A/B) 746 [single cone=710 (CKC=67; NETZ=71; LLETZ=572) repeat cones=36]<br>C) 170	A) 72899<br>B) 383<br>C) 170	National registries, hospital records, questionnaires	sPTB (<37w); sPTB (<37w)(single); sPTB (<37w)(repeat); sPTB (<37w)(singleton); sPTB (<32w); sPTB (<28w); pPROM (<37w); pPROM (<32w); pPROM (<28w); LBW (<2500g); LBW (<2000g); LBW (<1500g); PM; PM (<37w); PM (<32w); PM (<28w)	9

Study (Country)	Study Design	Comparison Group	Procedure	Treated*	Untreated*	Source of data	Outcomes	Newcastle-Ottawa score
Jones 1979 (UK)	Retrospective cohort (population-based)	External: matching for age, parity, social class, delivery date, singleton birth	CKC	66	264	Clinical records from Cardiff Cervical Cytology Study - Cardiff Birth Survey (registry)	PTB (<37w); PTB (<37w)(singleton); sPTB (<37w); CS; ID; PrecL (<2h); ProlL (>12h); LBW (<2500g); PM; SB	9
van de Vijner 2010 (Belgium)	Retrospective cohort (hospital-based)	External: matching for age, parity, year of delivery	Excision NOS (LC, LLETZ)	55 (LC=5; LLETZ=50)	55	Hospital records and questionnaires	PTB (<37w); PTB (<37w)(single); PTB (<37w)(repeat); PTB (<37w)(singleton); PTB (<37w)(multiple); PTB (<34w); tPTL; pPROM; CS; ID; IoL; oxytocin; LBW (<2500g); NICU; PM; SB	7
Werner 2010 (USA)	Retrospective cohort (hospital-based)	A) External<br>B) Internal (pre-treatment pregnancies)<br>Both regression for age, parity, race	LLETZ	551	A) 240348<br>B) 842	Hospital records	PTB (<37w); PTB (nulliparous)(<37w); PTB (singleton)(<37w); sPTB (<37w); pPROM; PM; SB	9
Andia 2011 (Spain)	Retrospective, cohort (population-based)	A) External<br>B) Internal (pre-treatment pregnancies)<br>Both regression for age, parity, smoking	LLETZ	189	A) 189<br>B) 189	Hospital records and registries	PTB (<37w); PTB (<37w)(nulliparous); PTB (<37w)(parous); PTB (<37w)(singleton); PTB (<35w); PTB (<32w); CS; LBW (<2500g); LBW (1500g)	9
Armarnik 2011 (Israel)	Retrospective cohort (hospital-based)	External: regression for age, birth order, year of delivery, smoking, cervical cerclage	Excision NOS (CKC, LC, LLETZ, other)	53	104617	Hospital records	PTB (<34w); CS; epidural; cerclage; PM	9
Lima 2011 (Portugal)	Retrospective cohort (hospital-based)	External: no matching, no regression	LC; LLETZ	29 (LC= 11; LLETZ=18)	58	Hospital records	PTB (<37w); PTB (<37w)(D≤10mm); PTB (<37w)(D>10mm); CS; LBW (<2500g); Apgar (<7)(5min)	7

Study (Country)	Study Design	Comparison Group	Procedure	Treated*	Untreated*	Source of data	Outcomes	Newcastle-Ottawa score
Jones 1979 (UK)	Retrospective cohort (population-based)	External: matching for age, parity, social class, delivery date, singleton birth	CKC	66	264	Clinical records from Cardiff Cervical Cytology Study - Cardiff Birth Survey (registry)	PTB (<37w); PTB (<37w)(singleton); sPTB (<37w); CS; ID; PrecL (<2h); ProlL (>12h); LBW (<2500g); PM; SB	9
Castanon 2012 (& 2014) (UK)	Retrospective cohort (hospital-based)	A) External (general population)<br>B) Biopsy no treatment<br>C) Internal (pre-treatment pregnancies)<br>D) Internal (self-matching)	Excision NOS (CKC, LC, LLETZ, other)	4776	A) 510660<br>B) 7263<br>C) 1173<br>D) 372	Hospital records and national registries	PTB (<37w); PTB (<37w)(D<10mm); PTB (<37w)(D≥10mm); PTB (<37w)(singleton); PTB (<33w)	8
Poon 2012 (UK)	Prospective cohort (hospital-based)	External: regression for parity, race, smoking, cervical length, PTB, miscarriage, LLETZ	LLETZ	473	25772	Hospital records, private practice records, questionnaires	sPTB (<37w); sPTB (<34w)	8
Reilly 2012 (UK)	Retrospective cohort (population-based)	A) External negative smear<br>B) Colposcopy +/- biopsy<br>Both regression for age, social deprivation, smoking, time to conception, obstetric history	Excision NOS (CKC, LLETZ); Ablation NOS (LA, CC, CT)	2162 (single excision=1546; single ablation=53; multiple=82)	A) 38983<br>B) 2534	National registries	PTB (<37w); PTB (<37w)(single); PTB (<37w)(repeat); PTB (<37w)(singleton); PTB (<32w); PTB (<28w); LBW (<2500g)	9

Study (Country)	Study Design	Comparison Group	Procedure	Treated*	Untreated*	Source of data	Outcomes	Newcastle-Ottawa score
Jones 1979 (UK)	Retrospective cohort (population-based)	External: matching for age, parity, social class, delivery date, singleton birth	CKC	66	264	Clinical records from Cardiff Cervical Cytology Study - Cardiff Birth Survey (registry)	PTB (<37w); PTB (<37w)(singleton); sPTB (<37w); CS; ID; PrecL (<2h); ProlL (>12h); LBW (<2500g); PM; SB	9
Simoens 2012 (Belgium)	Prospective cohort (hospital-based)	External: matching for hospital; regression for age, parity, ethnicity, smoking, education, HIV	LC; LLETZ; Excision NOS (CKC, LC, LLETZ) +/- Ablation NOS (LA, CC, CT)	97 [Excision=81 (CKC=8; LC=24; LLETZ=53; unknown=4); Ablation=8 (LA=6; CC=1; CT=1); both=8]	194	Hospital records; questionnaires and medical records	PTB (<37w); PTB (<37w)(D≤10mm); PTB (<37w)(D>10mm); PTB (<37w)(singleton); PTB (<32w); sPTB (<37w); sPTB (<32w); CS; LBW (<2500g)	9
Van Hentenryck 2012 (Belgium)	Retrospective cohort (hospital-based)	External: matching for age, parity, smoking, HIV	Excision NOS (CKC, LC, LLETZ)	106	212	Hospital records	PTB (<37w); PTB (<34w); tPTL; pPROM; chorioamnionitis; CS; ID; IoL; LBW (<2500g); NICU	9
Frega 2013 (Italy)	Prospective cohort (population-based)	External: matching for parity (nulliparous only), race (white only)	LLETZ	406	379	Hospital records	PTB (<37w); PTB (<37w)(nulliparous); PTB (<37w)(single); PTB (<37w)(singleton)	9
Frey 2013 (USA)	Retrospective cohort (hospital-based)	A) External with smear<br>B) Biopsy but no treatment<br>matching for age, year of treatment; regression for age, parity, race, diabetes, BMI, birth weight, CS	LLETZ	598	A) 588<br>B) 552	Hospital records and structured phone interviews	PTB (<37w); CS; IoL	8

Study (Country)	Study Design	Comparison Group	Procedure	Treated*	Untreated*	Source of data	Outcomes	Newcastle-Ottawa score
Jones 1979 (UK)	Retrospective cohort (population-based)	External: matching for age, parity, social class, delivery date, singleton birth	CKC	66	264	Clinical records from Cardiff Cervical Cytology Study - Cardiff Birth Survey (registry)	PTB (<37w); PTB (<37w)(singleton); sPTB (<37w); CS; ID; PrecL (<2h); ProlL (>12h); LBW (<2500g); PM; SB	9
Heinonen 2013 (Finland)	Retrospective cohort (population-based)	External: regression for age, socioeconomic status, marital status, urbanism, time to conception, PTB	LLETZ	7636	658179	National registers	PTB (<37w); PTB (<37w)(single); PTB (<37w)(repeat); PTB (<37w)(singleton)	9
Guo 2013 (China)	Prospective cohort (hospital-based)	Biopsy +/- CIN but no treatment: matching for smoking (non-smokers only)	CKC; LLETZ	84 (CKC=36; LLETZ=48)	68	Hospital records	PTB (<37w); PTB (<37w)(single); PTB (<34w); pPROM; CS; PrecL (<2h); ProlL (>12h); LBW (<2500g); Apgar (<7)(1min)	8
Wuntakal 2013 (UK)	Retrospective cohort (hospital-based)	A) Biopsy but no treatment<br>B) Internal, (pre-treatment pregnancies)<br>Both regression for parity, ethnicity, deprivation	Excision NOS (CKC, LC, LLETZ)	261	A) 257<br>B) 181	Hospital records	PTB (<37w); PTB (<37w)(single); PTB (<37w)(repeat); PTB (<33w); pPROM; CS; ID; LBW (<2500g)	9
Ciavattini 2014 (Italy)	Retrospective cohort (hospital-based)	External: matching for age, parity, BMI, smoking, hormonal contraception, PTB, cervical incompetence	LLETZ	7	21	Hospital records	sPTB (<36w)(multiple)	8

Study (Country)	Study Design	Comparison Group	Procedure	Treated*	Untreated*	Source of data	Outcomes	Newcastle-Ottawa score
Jones 1979 (UK)	Retrospective cohort (population-based)	External: matching for age, parity, social class, delivery date, singleton birth	CKC	66	264	Clinical records from Cardiff Cervical Cytology Study - Cardiff Birth Survey (registry)	PTB (<37w); PTB (<37w)(singleton); sPTB (<37w); CS; ID; PrecL (<2h); ProlL (>12h); LBW (<2500g); PM; SB	9
Ehsanipoor 2014 (USA)	Retrospective cohort (hospital-based)	External: regression for age, parity, race, PTB, smoking, drug use, chorionicity	CKC; LLETZ; Ablation NOS (LA, CT)	110 (CKC=10; LLETZ=36; Ablation NOS=64)	766	Hospital records	PTB (<37w)(multiple); PTB (<34w)(multiple); PTB (<28w)(multiple)	9
Kitson 2014 (UK)	Retrospective cohort (hospital-based)	Biopsy but no treatment: matching for age, parity, smoking	LLETZ	278	278	Hospital records	PTB (<37w); PTB (<37w)(singleton); PTB (<34w); sPTB; pPROM; CS; ID; LBW (<2500g); NICU	9
Sozen 2014 (Turkey)	Retrospective cohort (hospital-based)	External: matching for age, parity, obstetric history	CKC	15	24	Hospital records	PTB (<37w); pPROM; NICU	9
Martyn 2015 (Ireland)	Retrospective cohort (hospital-based)	Colposcopy but no treatment: matching for age	LLETZ; Excision NOS (CKC, repeat LLETZ)	297 (LLETZ=278; Excision NOS=19)	204	Hospital records and postal questionnaires	PTB (<37w); PTB (<37w)(single)	8
Stout 2015 (USA)	Retrospective cohort (hospital-based)	A) Cytology/biopsy but no treatment: matching for age, hospital, year<br>B) Internal (pre-treatment pregnancies)	LLETZ	598	A) 1129<br>B) 598	Hospital records and structured phone interviews	sPTB (<37w); sPTB (<37w)(singleton); sPTB (<34w)	9
Kirn 2015 (Germany)	Retrospective cohort (hospital-based)	External: matching for age, parity, smoking	Conization NOS	135	135	Hospital records	PTB (<37w); PTB (<37w)(singleton); CS	9

Study (Country)	Study Design	Comparison Group	Procedure	Treated*	Untreated*	Source of data	Outcomes	Newcastle-Ottawa score
Jones 1979 (UK)	Retrospective cohort (population-based)	External: matching for age, parity, social class, delivery date, singleton birth	CKC	66	264	Clinical records from Cardiff Cervical Cytology Study - Cardiff Birth Survey (registry)	PTB (<37w); PTB (<37w)(singleton); sPTB (<37w); CS; ID; PrecL (<2h); ProlL (>12h); LBW (<2500g); PM; SB	9
Miller 2015 (USA)	Retrospective cohort (hospital-based)	A) External<br>B) Women with untreated dysplasia<br>Both regression for age, body mass index at delivery, race/ethnicity, prior dysplasia, cervical length during pregnancy	Excision NOS	1356	A) 14149<br>B) 3023	Hospital records	PTB (<37w); PTB (<37w)(singleton)	9

*Numbers refer to women or pregnancies

APH: antepartum haemorrhage; BMI: body mass index; CC: cold coagulation; CIN: cervical intraepithelial neoplasia; CKC: cold knife conisation; CS: caesarean section; CT: cryotherapy; D: depth; HSIL: high-grade squamous intraepithelial lesion; ID: instrumental deliveries (ventouse/forceps); IoL: induction of labour; LA: laser ablation; LBW: low birthweight; LC: laser conisation; LLETZ: large loop excision of the transformation zone; MOH: massive obstetric haemorrhage; NETZ: needle excision of the transformation zone; NICU: neonatal intensive care unit admission; NOS: not otherwise specified; PM: perinatal mortality; PPH: postpartum haemorrhage; pPROM: preterm premature rupture of membranes; PreL: precipitous labour; ProlL: prolonged labour; PTB: preterm birth; RD: radical diathermy; SB: stillbirth; sPTB: spontaneous preterm birth; (s)PTB (single): (spontaneous) preterm birth (single cone); (s)PTB (repeat): (spontaneous) preterm birth (repeat cones); (s)PTB (singleton): (spontaneous) preterm birth (singleton pregnancies); (s)PTB (multiple): (spontaneous) preterm birth (multiple pregnancies); TOP: termination of pregnancy; tPTL: threatened preterm labour; USS: ultrasound scan;

Table B: Newcastle-Ottawa quality assessment of the included studies

		Selection				Comparability	Outcome		
Reference	Score	Representativeness of the exposed cohort	Selection of the non-exposed cohort	Ascertainment of exposure	Demonstration that outcome of interest was not present at start of study	Comparability of cohorts on the basis of the design or analysis	Assessment of outcome	Was follow-up long enough for outcomes to occur?	Adequacy of follow up of cohorts
Jones 1979	9	*Truly representative of the average pregnant woman with a previous history of treatment for CIN in the community	*drawn from the same community as the exposed cohort	*Secure record - hospital records	*Yes	**External: matching for age, parity, social class, date of delivery and singleton birth	*Record linkage	*Yes - retrospective	*Complete follow up - retrospective
Weber 1979	8	*Somewhat representative of the average pregnant woman with a previous history of treatment for CIN in the community	*drawn from the same community as the exposed cohort	*Structured interview	*Yes	*External: matching for age	*Record linkage	*Yes - retrospective	*Complete follow-up – retrospective
Buller 1982	7	*Somewhat representative of the average pregnant woman with a previous history of treatment for CIN in the community	*Internal (pre-treatment pregnancies)	*Secure record - hospital records	*Yes	*Internal (pre-treatment pregnancies)	*Record linkage	*Yes - retrospective	Inadequate: 27% lost to follow-up – no description of those lost
Hemmingsson 1982	8	*Somewhat representative of the average pregnant woman with a previous history of treatment for CIN in the community	*Internal (pre-treatment pregnancies)	*Secure record - hospital records	*Yes	*Internal (pre-treatment pregnancies)	*Record linkage	*Yes - retrospective	*Complete follow-up – retrospective
Larsson 1982	9	*Truly representative of the average pregnant woman with a previous history of treatment for CIN in the community	*Internal (pre-treatment pregnancies)	*Secure record - registry	*Yes	**Internal (pre-treatment pregnancies) with matching for age, parity, socioeconomic status, smoking, surgical interventions and various diseases	*Record linkage	*Yes – retrospective	*Complete follow-up – retrospective

		Selection				Comparability	Outcome		
Reference	Score	Representativeness of the exposed cohort	Selection of the non-exposed cohort	Ascertainment of exposure	Demonstration that outcome of interest was not present at start of study	Comparability of cohorts on the basis of the design or analysis	Assessment of outcome	Was follow-up long enough for outcomes to occur?	Adequacy of follow up of cohorts
Ludviksson 1982	8	*Somewhat representative of the average pregnant woman with a previous history of treatment for CIN in the community.	no description of the derivation of the non exposed cohort	*Secure record - hospital records	*Yes	**External: matching for age, parity and time of delivery	*Record linkage	*Yes - retrospective	*Complete follow up - retrospective
Moinian 1982	8	*Somewhat representative of the average pregnant woman with a previous history of treatment for CIN in the community	*Internal (pre-treatment pregnancies)	*Secure records – hospital records	*Yes	*Internal (pre-treatment pregnancies)	*Record linkage	*Yes - retrospective	*Complete follow up - retrospective
Anderson 1984	7	*Somewhat representative of the average pregnant woman with a previous history of treatment for CIN in the community	*drawn from the same community as the exposed cohort	*Secure record – hospital records	*Yes	**External: matching for age, race, births and miscarriages/TOP	Self-report	*Yes - retrospective	Inadequate: 25% lost to follow-up – no description of those lost
Kristensen 1985	9	*Truly representative of the average pregnant woman with a previous history of treatment for CIN in the community	*drawn from the same community as the exposed cohort	*Secure record - hospital records	*Yes	**External: matching for age and parity	*Record linkage (questionnaires for a minority that moved away)	*Yes - retrospective	*Complete follow up - retrospective
Kuoppala 1986	9	*Somewhat representative of the average pregnant woman with a previous history of treatment for CIN in the community	*drawn from the same community as the exposed cohort	*Secure record - hospital records	*Yes	**External: matching for age, parity and date of delivery	*Record-linkage	*Yes - retrospective	*Complete follow up - retrospective
Saunders 1986	6	*Somewhat representative of the average pregnant	*drawn from the same community as	Hospital case notes and contact with local general	*Yes	**External: matching for age, parity, race, year of delivery and	Hospital case notes and contact with local general	*Yes - retrospective	No description

Reference	Score	Selection				Comparability	Outcome		
		Representativeness of the exposed cohort	Selection of the non-exposed cohort	Ascertainment of exposure	Demonstration that outcome of interest was not present at start of study	Comparability of cohorts on the basis of the design or analysis	Assessment of outcome	Was follow-up long enough for outcomes to occur?	Adequacy of follow up of cohorts
		woman with a previous history of treatment for CIN in the community	the exposed cohort	practitioners		singleton pregnancy	practitioners		
Gunasekera 1992	9	*Somewhat representative of the average pregnant woman with a previous history of treatment for CIN in the community	*drawn from the same community as the exposed cohort	*Secure record- hospital records	*Yes	**External: matching for age, parity, race, duration of pregnancy and smoking habit	*Record linkage	*Yes- retrospective	*Complete follow up - retrospective
Blomfield 1993	9	*Somewhat representative of the average pregnant woman with a previous history of treatment for CIN in the community	*drawn from the same community as the exposed cohort	*Secure record – hospital records	*Yes	**External: matching for age, parity and ethnicity	*Record linkage	*Yes - retrospective	*Complete follow-up – retrospective
Haffenden 1993	9	*Somewhat representative of the average pregnant woman with a previous history of treatment for CIN in the community	*drawn from the same community as the exposed cohort	*Secure record - hospital records	*Yes	**External: matching for age and parity	*Record linkage	*Yes - retrospective	*Complete follow-up – retrospective
Hagen 1993	9	*Somewhat representative of the average pregnant woman with a previous history of treatment for CIN in the community.	*drawn from the same community as the exposed cohort	*Secure record - hospital records	*Yes	**External: matching for age and parity; regression analysis for maternal height, marital status, level of education, smoking, previous TOP, and, in the index pregnancy, occurrence of gestational hypertension or antepartum	*Record linkage	*Yes - retrospective	*Subjects lost to follow up (1.7%) unlikely to introduce bias

		Selection					Comparability	Outcome		
Reference	Score	Representativeness of the exposed cohort	Selection of the non-exposed cohort	Ascertainment of exposure	Demonstration that outcome of interest was not present at start of study	Comparability of cohorts on the basis of the design or analysis	Assessment of outcome	Was follow-up long enough for outcomes to occur?	Adequacy of follow up of cohorts
						haemorrhage and the mode of delivery			
Kristensen 1993	7	*Truly representative of the average pregnant woman with a previous history of treatment for CIN in the community	*A) External: drawn from the same community as the exposed cohort<br>B) Internal (self-matching)	*Secure record - registry	*Yes	A) External: no matching, no regression analysis<br>B) Internal (self-matching)	*Record linkage	*Yes - retrospective	*Complete follow-up – retrospective
Braet 1994	9	*Somewhat representative of the average pregnant woman with a previous history of treatment for CIN in the community	*drawn from the same community as the exposed cohort	*Secure record - hospital records	*Yes	**External: matching for age, parity and smoking	*Record linkage	*Yes - retrospective	*Complete follow-up – retrospective
Cruickshank 1995	7	*Somewhat representative of the average pregnant woman with a previous history of treatment for CIN in the community	*A) External: drawn from the same community as the exposed cohort<br>B) Internal (pre-treatment pregnancies)	*Secure record – registry	*Yes	**A) External: matching for maternal age, parity, husband's or partner's social class, height and daily cigarette consumption<br>B) Internal (pre-treatment pregnancies)	Record linkage but also self-report	*Yes - retrospective	Inadequate: 34.7% did not respond to the questionnaire – no description of those lost
Sagot 1995	7	*Somewhat representative of the average pregnant woman with a previous history of treatment for CIN in the community.	*Internal (pre-treatment pregnancies)	*Secure record - hospital records	*Yes	*Internal (pre-treatment pregnancies)	*Record linkage	*Yes - retrospective	Inadequate: 21.6% could not be recontacted – no description of those lost
Spitzer 1995	7	*Somewhat representative of the average pregnant woman with a previous	*Internal (pre-treatment pregnancies)	*Secure record – hospital/private practice records	*Yes	**Internal (pre-treatment pregnancies) with matching for age and parity	Self-report	*Yes - retrospective	Inadequate: 47.9% lost to follow-up – no description of

Reference	Score	Selection				Comparability	Outcome		
Reference	Score	Representativeness of the exposed cohort	Selection of the non-exposed cohort	Ascertainment of exposure	Demonstration that outcome of interest was not present at start of study	Comparability of cohorts on the basis of the design or analysis	Assessment of outcome	Was follow-up long enough for outcomes to occur?	Adequacy of follow up of cohorts
		history of treatment for CIN in the community							those lost
Bekassy 1996	8	*Somewhat representative of the average pregnant woman with a previous history of treatment for CIN in the community	A) External: drawn from a different source B) Internal (self-matching)	*Secure record - hospital records	*Yes	**A) External: matching for age, parity and time of delivery B) Internal (self-matching)	*Record linkage	*Yes - retrospective	*Complete follow up - retrospective
Forsmo 1996	8	*Somewhat representative of the average pregnant woman with a previous history of treatment for CIN in the community	*drawn from a same area & period but may be other institutions	*Secure record - hospital records	*Yes	**External: matching for age, parity and place of delivery	Self-report & record linkage for some outcomes	*Yes - retrospective	*Subjects lost to follow-up (3.4%) unlikely to introduce bias
Turlington 1996	7	*Somewhat representative of the average pregnant woman with a previous history of treatment for CIN in the community	*Drawn from the same community as the exposed cohort	*Secure record - hospital records	*Yes	**Women with colposcopically directed biopsy: regression analysis for age	Self-report	*Yes - retrospective	Inadequate: 29.7% did not respond - no description of those lost
Raio 1997	9	*Somewhat representative of the average pregnant woman with a previous history of treatment for CIN in the community	*A) External: drawn from the same community as the exposed cohort B) Internal (self-matching)	*Secure record - hospital records	*Yes	**A) External: matching for age, parity, marital status, social class, smoking habits and previous PTB B) Internal (self-matching)	*Record linkage	*Yes - retrospective	*Subjects lost to follow-up (11.4%) unlikely to introduce bias
Andersen 1999	9	*Somewhat representative of the average pregnant woman with a previous history of treatment	*Drawn from the same community as the exposed cohort	*Secure record - hospital records	*Yes	**External: matching for age and parity	*Record-linkage	*Yes - retrospective	*Complete follow up - retrospective

		Selection				Comparability	Outcome		
**Reference**	**Score**	**Representativeness of the exposed cohort**	**Selection of the non-exposed cohort**	**Ascertainment of exposure**	**Demonstration that outcome of interest was not present at start of study**	**Comparability of cohorts on the basis of the design or analysis**	**Assessment of outcome**	**Was follow-up long enough for outcomes to occur?**	**Adequacy of follow up of cohorts**
		for CIN in the community							
El-Bastawissi 1999	9	*Truly representative of the average pregnant woman with a previous history of treatment for CIS in the community	*Drawn from the same community as the exposed cohort	*Secure record – population-based cancer registry and birth certificates	*Yes	**A) External: matching for age and country of origin<br>B) Women with untreated HSIL: no matching<br>Both had regression analysis for parity, race, maternal smoking, marital status and history of TOPs	*Record linkage	*Yes - retrospective	*Complete follow up - retrospective
van Rooijen 1999	9	*Somewhat representative of the average pregnant woman with a previous history of treatment for CIN in the community	*Drawn from the same source as the treated group	*Secure record - hospital records	*yes	**External: matching for age, parity and year of delivery	*Record linkage	*Yes - retrospective	*Subjects lost to follow-up (16.5%) unlikely to introduce bias
Paraskevaidis 2002	9	*Somewhat representative of the average pregnant woman with a previous history of treatment for IA1 cervical carcinoma in the community	*drawn from the same community as the exposed cohort	*Secure record - hospital records	*Yes	**External: matching for age, parity, smoking, multiple pregnancies and history of previous PTBs	*Record linkage	*Yes - retrospective	*Complete follow up - retrospective
Sadler 2004	9	*Somewhat representative of the average pregnant woman with a previous history of treatment for CIN in the community	*Drawn from the same community as the exposed cohort	*Secure record - hospital records	*Yes	**Women with colposcopy: regression analysis for age, ethnicity, socioeconomic status, smoking in pregnancy, previous obstetric history, transfer to the National Women's	*Record linkage	*Yes - retrospective	*Complete follow-up – retrospective

		Selection				Comparability	Outcome		
Reference	Score	Representativeness of the exposed cohort	Selection of the non-exposed cohort	Ascertainment of exposure	Demonstration that outcome of interest was not present at start of study	Comparability of cohorts on the basis of the design or analysis	Assessment of outcome	Was follow-up long enough for outcomes to occur?	Adequacy of follow up of cohorts
						Hospital and antepartum hemorrhage			
Tan 2004	8	*Somewhat representative of the average woman with CIN in the community	*Drawn from the same community as the exposed cohort	*Secure record - hospital records	*Yes	**External: matching for age and parity	*Record linkage	*Yes - retrospective	Inadequate: in 29.7% incomplete retrieval of data
Acharya 2005	9	*Somewhat representative of the average pregnant woman with a previous history of treatment for CIN in the community	*A) External: drawn from the same community as the exposed cohort<br>B) Internal (pre-treatment pregnancies)	*Secure record – hospital records	*Yes	**A) External: matching for age, parity, date of delivery, smoking and previous obstetric history<br>B) Internal (pre-treatment pregnancies)	*Record linkage	*Yes - retrospective	*Complete follow-up - retrospective
Samson 2005	9	*Somewhat representative of the average pregnant woman with a previous history of treatment for CIN in the community	*Drawn from the same community as the exposed cohort	*Secure record – official databases	*Yes	**External: matching for age, parity, smoking status, year of delivery	*Record linkage	*Yes - retrospective	*Complete follow-up – retrospective
Crane 2006	8	*Somewhat representative of the average pregnant woman with a previous history of treatment for CIN in the community	*Drawn from the same community as the exposed cohort	no description	*Yes	**External: regression analysis for maternal age, gestational age at the time of transvaginal ultrasonography, parity, smoking, antepartum bleeding after 20 weeks of gestation and previous sPTB	*Record-linkage	*Yes - retrospective	*Complete follow-up – retrospective
Klaritsch 2006	7	*Somewhat representative of the	*Drawn from the same	*Secure record - hospital records	*Yes	External: no matching, no regression analysis	*Record linkage	*Yes - retrospective	*Complete follow-up –

		Selection				Comparability	Outcome		
**Reference**	**Score**	**Representativeness of the exposed cohort**	**Selection of the non-exposed cohort**	**Ascertainment of exposure**	**Demonstration that outcome of interest was not present at start of study**	**Comparability of cohorts on the basis of the design or analysis**	**Assessment of outcome**	**Was follow-up long enough for outcomes to occur?**	**Adequacy of follow up of cohorts**
		average pregnant woman with a previous history of treatment for CIN in the community	community as the exposed cohort						retrospective
Bruinsma 2007	9	*Somewhat representative of the average pregnant woman with a previous history of treatment for CIN in the community	*Drawn from the same community as the exposed cohort	*Secure record - hospital records	*Yes	**Women with colposcopy but no treatment: regression analysis for for age, illicit drug use during pregnancy, delivery at the RWH, marital status, maternal medical condition, previous TOP, previous miscarriage, previous PTB and previous treatment	*Record linkage	*Yes - retrospective	*Complete follow-up – retrospective
Himes 2007	8	*Somewhat representative of the average pregnant woman with a previous history of treatment for CIN in the community	*Drawn from the same community as the exposed cohort	*Secure record – hospital records	*Yes	*Women with colposcopic biopsy but no treatment – no matching, no regression analysis	*Record linkage	*Yes - retrospective	*Complete follow-up – retrospective

		Selection				Comparability	Outcome		
**Reference**	**Score**	**Representativeness of the exposed cohort**	**Selection of the non-exposed cohort**	**Ascertainment of exposure**	**Demonstration that outcome of interest was not present at start of study**	**Comparability of cohorts on the basis of the design or analysis**	**Assessment of outcome**	**Was follow-up long enough for outcomes to occur?**	**Adequacy of follow up of cohorts**
Jakobsson 2007	9	*Truly representative of the average pregnant woman with a previous history of treatment for CIN in the community	*Drawn from the same community as the exposed cohort	*Secure record – national registers	*Yes	**External: regression analysis for age, parity and smoking	*Record linkage	*Yes - retrospective	*Complete follow-up – retrospective
Sjoborg 2007	8	*Truly representative of the average pregnant woman with a previous history of treatment for CIN in the community	*A) External: drawn from the same community as the exposed cohort<br>B) Internal (self-matching)	*Secure record – hospital records	*Yes	**A) External: matching for age, parity and plurality<br>B) Internal (self-matching)<br>Both had regression analysis for smoking, marital status and education	*Record linkage	*Yes - retrospective	Inadequate: 69% of the women did not respond or did not give their consent – no description of those lost
Albrechtesen 2008	9	*Truly representative of the average pregnant woman with a previous history of treatment for CIN in the community	*A) External: drawn from the same community as the exposed cohort<br>B) Internal (pre-treatment pregnancies)	*Secure record - national registries	*Yes	**A) External<br>B) Internal (pre-treatment pregnancies)<br>Both had regression analysis for age and birth order	*Record linkage	*Yes - retrospective	*Complete follow-up – retrospective
Parikh 2008	6	*Somewhat representative of the average pregnant woman with a previous history of treatment for CIN in the community	*Drawn from the same community as the exposed cohort	No description	*Yes	External: No matching, no regression analysis	*Record linkage	*Yes - retrospective	*Subjects lost to follow-up (10.3%) unlikely to introduce bias

		Selection				Comparability	Outcome		
**Reference**	**Score**	**Representativeness of the exposed cohort**	**Selection of the non-exposed cohort**	**Ascertainment of exposure**	**Demonstration that outcome of interest was not present at start of study**	**Comparability of cohorts on the basis of the design or analysis**	**Assessment of outcome**	**Was follow-up long enough for outcomes to occur?**	**Adequacy of follow up of cohorts**
Jakobsson 2009	8	*Somewhat representative of the average pregnant woman with a previous history of treatment for CIN in the community	A) External: treated group drawn from hospital while controls from population-based registry<br>B) Internal (self-matching)	*Secure record – national registers and hospital records	*Yes	**A) External: no matching<br>B) Internal (self-matching)<br>Both had regression analysis for age, parity, or both	*Record linkage	*Yes - retrospective	*Complete follow-up – retrospective
Noehr 2009 (singletons & cone depth)	9	*Truly representative of the average pregnant woman with a previous history of treatment for CIN in the community	*Drawn from the same community as the exposed cohort	*Secure record – national registries	*Yes	**A) External<br>B) Women with biopsy but no treatment<br>Both had regression analysis for age, year of delivery, smoking during pregnancy and marital status during pregnancy	*Record linkage	*Yes - retrospective	*Complete follow-up - retrospective
Noehr 2009 (twins)	9	*Truly representative of the average pregnant woman with a previous history of treatment for CIN in the community	*Drawn from the same community as the exposed cohort	*Secure record – national registries	*Yes	**External: regression analysis for age, year of delivery, smoking during pregnancy, marital status during pregnancy and IVF	*Record linkage	*Yes - retrospective	*Complete follow-up - retrospective
Shanbhag 2009	8	*Truly representative of the average pregnant woman with a previous history of treatment for CIN3 in the community	*Drawn from the same community as the exposed cohort	*Secure record – national registries	*Yes	**A) External<br>B) Women with untreated CIN 3<br>Both had regression analysis for maternal age at delivery, smoking, socioeconomic status, year of delivery, birth weight, malpresentation, sPTB and pPROM	*Record linkage	*Yes - retrospective	Inadequate: for 69% of the treated population the type of treatment was not known – no description of those lost
Fischer 2010	8	*Somewhat	*Drawn from	No description	*Yes	**External: regression	*Record linkage	*Yes	*Complete

		Selection				Comparability	Outcome		
**Reference**	**Score**	**Representativeness of the exposed cohort**	**Selection of the non-exposed cohort**	**Ascertainment of exposure**	**Demonstration that outcome of interest was not present at start of study**	**Comparability of cohorts on the basis of the design or analysis**	**Assessment of outcome**	**Was follow-up long enough for outcomes to occur?**	**Adequacy of follow up of cohorts**
		representative of the average pregnant woman with a previous history of treatment for CIN in the community	the same community as the exposed cohort			analysis for age, race, the number of prior vaginal deliveries at ≥20 weeks and gestational age at the time of cervical sonography			follow-up
Ortoft 2010	9	*Somewhat representative of the average pregnant woman with a previous history of treatment for CIN in the community	*A) External<br>B) Women with untreated HSIL<br>Both were drawn from the same community as the exposed cohort<br>C) Internal (self-matching)	*Secure record – national registries	*Yes	** A) External<br>B) Women with untreated HSIL<br>Both had regression analysis for age, parity, smoking status, educational level and marital status<br>C) Internal (self-matching)	*Record linkage (but questionnaires for the outcomes of previous pregnancies when internal matching (self-matching) was used)	*Yes - retrospective	*Complete follow-up
van de Vijner 2010	7	*Somewhat representative of the average pregnant woman with a previous history of treatment for CIN in the community	*Drawn from the same community as the exposed cohort	*Secure record – hospital records	*Yes	**External: matching for age, parity and year of delivery	Self-report	*Yes - retrospective	No statement
Werner 2010	9	*Somewhat representative of the average pregnant woman with a previous history of treatment for CIN in the community	*A) External: drawn from the same community as the exposed cohort<br>B) Internal (pre-treatment pregnancies)	*Secure record – hospital records	*Yes	**A) External<br>B) Internal (pre-treatment pregnancies)<br>Both had regression analysis for age, parity and race	*Record linkage	*Yes - retrospective	*Complete follow-up - retrospective
Andia 2011	9	*Truly representative of the average pregnant woman with a previous history of	*A) External: drawn from the same community as	*Secure record – hospital records	*Yes	**A) External<br>B) Internal (pre-treatment pregnancies)<br>Both had regression	*Record linkage	*Yes - retrospective	*Complete follow-up - retrospective

		Selection				Comparability	Outcome		
**Reference**	**Score**	**Representativeness of the exposed cohort**	**Selection of the non-exposed cohort**	**Ascertainment of exposure**	**Demonstration that outcome of interest was not present at start of study**	**Comparability of cohorts on the basis of the design or analysis**	**Assessment of outcome**	**Was follow-up long enough for outcomes to occur?**	**Adequacy of follow up of cohorts**
		treatment for CIN in the community	the exposed cohort<br>B) Internal (pre-treatment pregnancies)			analysis for age, parity and smoking			
Armarnik 2011	9	*Somewhat representative of the average pregnant women with a previous history of treatment for CIN in the community	*Drawn from the same community as the exposed cohort	*Secure record – hospital records	*Yes	**External: regression analysis for age, birth order, year of delivery, smoking and cervical incompetence with cerclage	*Record linkage	*Yes - retrospective	*Subjects lost to follow-up (7%) unlikely to introduce bias
Lima 2011	7	*Somewhat representative of the average pregnant woman with a previous history of treatment for CIN in the community	*Drawn from the same community as the exposed cohort	*Secure record – hospital records	*Yes	No matching, no regression analysis	*Record linkage	*Yes - retrospective	*Complete follow-up – retrospective
Castanon 2012 (& 2014)	8	*Somewhat representative of the average pregnant woman with a previous history of treatment for CIN in the community	*A) External (general population)<br>B) Women with punch biopsy<br>C) Internal (pre-treatment pregnancies)<br>D) Internal matching (self-matching)	*Secure record – hospital records	*Yes	**A) General population<br>B) Women with punch biopsy<br>C/D) Internal controls Regression analysis for age parity and study site for a variant of the groups that we used	*Record linkage	*Yes - retrospective	Inadequate: 29.9% lost to follow-up because of unknown gestational age – no description of those lost
Poon 2012	8	*Somewhat representative of the average pregnant woman with a previous history of treatment for CIN in the community	*Drawn from the same community as the exposed cohort	Written self-report (questionnaires)	*Yes	**External: regression analysis for parity, race, smoking, cervical length, previous delivery at term, previous PTB, previous miscarriage and	*Record linkage	*Yes - retrospective	*Complete follow-up - retrospective

		Selection				Comparability	Outcome		
Reference	Score	Representativeness of the exposed cohort	Selection of the non-exposed cohort	Ascertainment of exposure	Demonstration that outcome of interest was not present at start of study	Comparability of cohorts on the basis of the design or analysis	Assessment of outcome	Was follow-up long enough for outcomes to occur?	Adequacy of follow up of cohorts
						previous LLETZ (for the prediction of sPTB)			
Reilly 2012	9	*Truly representative of the average pregnant woman with a previous history of treatment for CIN in the community	*Drawn from the same community as the exposed cohort	*Secure record – national registries	*Yes	**A) External<br>B) Women with colposcopy +/- punch biopsy<br>Both had regression analysis for maternal age at birth, social deprivation, smoking status, time interval between screening/colposcopy/treatment and conception, any history of a previous adverse pregnancy outcome (and gestational age for LBW outcome)	*Record linkage	*Yes - retrospective	*Subjects lost to follow-up (10.6%) unlikely to introduce bias
Simoens 2012	9	*Somewhat representative of the average pregnant woman with a previous history of treatment for CIN in the community	*Drawn from the same community as the exposed cohort	*Secure record – questionnaires in combination with checking of medical files	*Yes	**External: matching for admittance in the same maternity ward; regression analysis for age, parity, ethnicity, smoking, education, HIV status	*Record linkage	*Yes	*Complete follow-up
Van Hentenryck 2012	9	*Somewhat representative of the average pregnant woman with a previous history of treatment for CIN in the community	*Drawn from the same community as the exposed cohort	*Secure record – hospital records	*Yes	**External: matching for age at delivery, parity, smoking, history of gestation and HIV status	*Record linkage	*Yes - retrospective	*Complete follow-up - retrospective
Frega 2013	9	*Truly representative of the average pregnant woman with a previous history of	*Drawn from the same community as the exposed	*Secure record - hospital records	*Yes	**External: women of the same parity (only nulliparous) and race (only white)	*Record linkage	*Yes	*Subjects lost to follow up (4.1%) unlikely to introduce bias

		Selection				Comparability	Outcome		
**Reference**	**Score**	**Representativeness of the exposed cohort**	**Selection of the non-exposed cohort**	**Ascertainment of exposure**	**Demonstration that outcome of interest was not present at start of study**	**Comparability of cohorts on the basis of the design or analysis**	**Assessment of outcome**	**Was follow-up long enough for outcomes to occur?**	**Adequacy of follow up of cohorts**
		treatment for CIN in the community	cohort						
Frey 2013	8	*Somewhat representative of the average pregnant woman with a previous history of treatment for CIN in the community	*Drawn from the same community as the exposed cohort	*Secure record – hospital records	*Yes	**A) External<br>B) Women with punch biopsy<br>Both had matching for age and year of treatment, and regression analysis for age, parity, race, maternal diabetes, maternal BMI, neonate birth weight and prior CS	*Record linkage (structured phone interviews and then confirmation from medical files)	*Yes - retrospective	No statement
Heinonen 2013	9	*Truly representative of the average pregnant woman with a previous history of treatment for CIN in the community	*Drawn from the same community as the exposed cohort	*Secure record – hospital records	*Yes	**External: regression analysis for maternal age, socioeconomic status, marital status, urbanism, time since LLETZ and previous PTBs	*Record linkage	*Yes - retrospective	*Complete follow-up - retrospective
Guo 2013	8	*Somewhat representative of the average pregnant woman with a previous history of treatment for CIN in the community	*Drawn from the same community as the exposed cohort	*Secure record – hospital records	*Yes	**Women with colposcopic biopsy +/- CIN: all were non-smokers	*Record linkage	*Yes	No statement
Wuntakal 2013	9	*Somewhat representative of the average pregnant woman with a previous history of treatment for CIN in the community	*A) Women with biopsy: drawn from the same community as the exposed cohort<br>B) Internal (pre-treatment	*Secure record – hospital records	*Yes	**A) Women with biopsy<br>B) Internal (pre-treatment pregnancies)<br>Both had regression analysis for parity, ethnicity and deprivation	*Record linkage	*Yes - retrospective	*Complete follow-up - retrospective

		Selection				Comparability	Outcome		
**Reference**	**Score**	**Representativeness of the exposed cohort**	**Selection of the non-exposed cohort**	**Ascertainment of exposure**	**Demonstration that outcome of interest was not present at start of study**	**Comparability of cohorts on the basis of the design or analysis**	**Assessment of outcome**	**Was follow-up long enough for outcomes to occur?**	**Adequacy of follow up of cohorts**
			pregnancies)						
Ciavattini 2014	8	Selected group of users (twin deliveries after assisted reproduction techniques)	*Drawn from the same community as the exposed cohort	*Secure record - hospital records	*Yes	**External: matching for age, parity, BMI, tabagism, previous hormonal contraception, previous PTB and cervical incompetence at 1st trimester	*Record linkage	*Yes - retrospective	*Complete follow-up - retrospective
Ehsanipoor 2014	9	*Somewhat representative of the average pregnant woman (with a twin pregnancy) with a previous history of treatment for CIN in the community	*Drawn from the same community as the exposed cohort	*Secure record – hospital records	*Yes	**External: regression analysis for age, parity, race, history of PTB, history of tobacco use, history of drug use and chorionicity	*Record linkage	*Yes - retrospective	*Complete follow-up - retrospective
Kitson 2014	9	*Somewhat representative of the average pregnant woman with a previous history of treatment for CIN in the community	*Drawn from the same community as the exposed cohort	*Secure record – hospital records	*Yes	**Women with punch biopsy: matching for age, parity and smoking	*Record linkage	*Yes - retrospective	*Complete follow-up - retrospective
Sozen 2014	9	*Somewhat representative of the average pregnant woman with a previous history of treatment for CIN in the community	*Drawn from the same community as the exposed cohort	*Secure record – hospital records	*Yes	**External: matching for age, parity and obstetric history	*Record linkage	*Yes - retrospective	*Complete follow up - retrospective
Martyn 2015	8	*Somewhat representative of the average pregnant woman with a previous history of treatment for CIN in the	*Drawn from the same community as the exposed cohort	*Secure record - questionnaires which were then confirmed from hospital records	*Yes	**Women with colposcopy: matching for age	Self-report	*Yes - retrospective	*Complete follow up - retrospective

		Selection				Comparability	Outcome		
**Reference**	**Score**	**Representativeness of the exposed cohort**	**Selection of the non-exposed cohort**	**Ascertainment of exposure**	**Demonstration that outcome of interest was not present at start of study**	**Comparability of cohorts on the basis of the design or analysis**	**Assessment of outcome**	**Was follow-up long enough for outcomes to occur?**	**Adequacy of follow up of cohorts**
		community							
Stout 2015	9	*Somewhat representative of the average pregnant woman with a previous history of treatment for CIN in the community	*A) Women with cervical cytology/punch biopsy: drawn from the same community as the exposed cohort<br>B) Internal (pre-treatment pregnancies)	*Secure record – hospital records	*Yes	**A) Women with cervical cytology/punch biopsy: matching for age, hospital site and calendar year of cervical procedure<br>B) Internal (pre-treatment pregnancies)	*Structured phone interviews which were then confirmed from medical files	**Yes - retrospective	*Subjects lost to follow up (<6%) unlikely to introduce bias
Kirn 2015	9	*Somewhat representative of the average pregnant woman with a previous history of treatment for CIN in the community	*drawn from the same community as the exposed cohort	*Secure record - hospital records	*Yes	**External: matching for age, parity, smoking	*Record linkage	*Yes - retrospective	*Complete follow up - retrospective
Miller 2015	9	*Somewhat representative of the average pregnant woman with a previous history of treatment for CIN in the community	*drawn from the same community as the exposed cohort	*Secure record - hospital records	*Yes	**A) External<br>B) Women with untreated dysplasia.<br>In both groups regression analysis adjusted for age, body mass index at delivery, race/ethnicity, prior dysplasia and cervical length during pregnancy	*Record linkage	*Yes - retrospective	*Complete follow-up – retrospective

Table C Preterm birth in women with cervical intraepithelial neoplasia (CIN) for treated versus untreated women according to number of fetuses*

Preterm birth outcome	No of studies	Total No of women	No (%) of women		Effect estimate RR (95% CI)	P value for heterogeneity (I^2%)
			Treated	Untreated		
**<37 weeks' gestation and singleton pregnancy**						
All treatment types	32	2 189 620	2907/33 330 (8.7)	110 981/2 156 290 (5.1)	1.76 (1.57 to 1.98)	<0.001 (78)
CKC	6	37 759	83/495 (16.8)	2286/37 264 (6.1)	2.89 (2.22 to 3.77)	0.62 (0)
LC	4	545	52/249 (20.9)	24/296 (8.1)	2.54 (1.24 to 5.2)	0.08 (55)
NETZ	1	7399	17/71 (23.9)	301/7328 (4.1)	5.83 (3.80 to 8.95)	N/E
LLETZ	18	1 444 175	1660/20 812 (8.0)	66 533/1 423 363 (4.7)	1.61 (1.39 to 1.87)	<0.001 (76)
LA	3	3420	129/1325 (9.7)	188/2095 (9.0)	1.10 (0.75 to 1.62)	0.18 (42)
CT	1	58	1/36 (2.8)	0/22 (0.0)	1.86 (0.08 to 43.87)	N/E
RD	1	2150	109/760 (14.3)	123/1390 (8.8)	1.62 (1.27 to 2.06)	N/E
Excisional treatment NOS	6	542 622	713/7133 (10.0)	35 877/535 489 (6.7)	1.43 (1.15 to 1.77)	0.05 (56)
Ablative treatment NOS	2	110 091	99/2099 (4.7)	3670/107 992 (3.4)	1.14 (0.56 to 2.32)	0.2 (40)
Treatment NOS	3	41 401	44/350 (12.6)	1979/41 051 (4.8)	2.20 (1.28 to 3.78)	0.07 (62)
**<37 weeks' gestation and multiple pregnancy**						
All treatment types	6	10 825	138/299 (46.2)	3585/10 526 (34.1)	1.13 (0.95 to 1.34)	0.25 (23)
CKC	2	84	5/13 (38.5)	37/71 (52.1)	0.95 (0.49 to 1.83)	1 (0)
LLETZ	4	10 227	98/219 (44.7)	3308/10 008 (33.1)	1.26 (1.08 to 1.46)	0.44 (0)
Excisional treatment NOS	1	4	3/3 (100.0)	0/1 (0.0)	3.5 (0.31 to 39.71)	N/E
Ablative treatment NOS	1	510	32/64 (50.0)	240/446 (53.8)	0.93 (0.72 to 1.20)	N/E
**<32-34 weeks' gestation and multiple pregnancy**						
All treatment types	3	10 789	38/286 (13.3)	715/10 503 (6.8)	1.68 (0.95 to 2.98)	0.08 (52)
CKC	1	80	4/10 (40.0)	8/70 (11.4)	3.5 (1.29 to 9.52)	N/E
LLETZ	3	10 199	28/212 (13.2)	658/9987 (6.6)	1.76 (0.88 to 3.5)	0.21 (36)
Ablative treatment NOS	1	510	6/64 (9.4)	49/446 (11.0)	0.85 (0.38 to 1.91)	N/E
**<28 weeks' gestation and multiple pregnancy**						
All treatment types	2	10 744	12/276 (4.3)	237/10 468 (2.3)	2.43 (1.40 to 4.22)	0.88 (0)
CKC	1	80	0/10 (0.0)	1/70 (1.4)	2.15 (0.09 to 49.56)	N/E
LLETZ	2	10 154	10/202 (5.0)	230/9952 (2.3)	2.45 (1.34 to 4.47)	0.42 (0)
Ablative treatment NOS	1	510	2/64 (3.1)	6/446 (1.3)	2.32 (0.48 to 11.26)	N/E

CKC=cold knife conisation; CT=cryotherapy; LA=laser ablation; LC=laser conisation; LLETZ=large loop excision of transformation zone; N/E=not eligible; NETZ=needle excision of transformation zone; NOS=not otherwise specified; RD=radical diathermy.

*If study had more than one comparison groups, we used external groups (external general, external untreated women that had colposcopy+/−CIN+/−biopsy, women with HSIL but no treatment) in preference to internal comparators (self matching or pregnancies before treatment).

Table D: Preterm birth (<37 weeks) for treated versus treated women for various cone dimensions (depth/volume)

Comparison Group 1	Comparison Group 2	Studies	Total N	Treated n/N (%)	Untreated n/N (%)	Effect Estimate RR (95% CI)	Heterogeneityp-value (I^2%)
**Cone Depth**							
**Cone Depth ≥ 10-12mm**	**Cone Depth ≤ 10-12mm**						
All Treatment types	All Treatment types	7	6359	403/3276 (12.3)	239/3083 (7.8)	1.54 [1.31, 1.80]	0.48 (0)
LC	LC	1	64	5/23 (21.7)	1/41 (2.4)	8.91 [1.11, 71.73]	N/E (N/E)
LLETZ	LLETZ	2	836	25/258 (9.7)	44/578 (7.6)	1.26 [0.74, 2.17]	0.98 (0)
Excision NOS	Excision NOS	4	5459	373/2995 (12.5)	194/2464 (7.9)	1.55 [1.31, 1.83]	0.52 (0)
**Cone Depth ≥ 15-17mm**	**Cone Depth ≤ 15-17mm**						
All Treatment types	All Treatment types	4	4275	167/1661 (10.1)	149/2614 (5.7)	1.82 [1.47, 2.26]	0.55 (0)
LC	LC	1	75	14/61 (23.0)	0/14 (0)	7.02 [0.44, 111.1]	N/E (N/E)
LLETZ	LLETZ	2	3869	128/1499 (8.5)	117/2370 (4.9)	1.86 [1.36, 2.55]	0.28 (14)
Excisional Treatment NOS	Excisional Treatment NOS	1	331	25/101 (24.8)	32/230 (13.9)	1.78 [1.11, 2.84]	N/E (N/E)
**Cone Depth ≥ 20mm**	**Cone Depth ≤ 20mm**						
All Treatment types	All Treatment types	3	3944	87/851 (10.2)	174/3093 (5.6)	2.79 [1.24, 6.27]	0.06 (64)
LC	LC	1	75	12/42 (28.6)	2/33 (6.1)	4.71 [1.13, 19.62]	N/E (N/E)
LLETZ	LLETZ	2	3869	75/809 (9.3)	172/3060 (5.6)	2.47 [0.94, 6.51]	0.05 (74)
**Cone Depth ≥ 15-17mm**	**Cone Depth ≤ 10-12mm**						
All Treatment types	All Treatment types	3	2841	153/1600 (9.6)	76/1241 (6.1)	1.70 [1.31, 2.22]	0.52 (0)
LLETZ	LLETZ	2	2624	128/1499 (8.5)	62/1125 (5.5)	1.63 [1.21, 2.19]	0.36 (0)
Excisional Treatment NOS	Excisional Treatment NOS	1	217	25/101 (24.8)	14/116 (12.1)	2.05 [1.13, 3.73]	N/E (N/E)
**Cone Depth ≥ 20mm**	**Cone Depth ≤ 10-12mm**						
All Treatment types	All Treatment types	2	1934	75/809 (9.3)	62/1125 (5.5)	2.49 [0.93, 6.66]	0.08 (67)
LLETZ	LLETZ	2	1934	75/809 (9.3)	62/1125 (5.5)	2.49 [0.93, 6.66]	0.08 (67)
**Cone Depth ≥ 20mm**	**Cone Depth ≤ 15mm**						
All Treatment types	All Treatment types	3	3240	87/856 (10.2)	117/2384 (4.9)	3.07 [1.27, 7.45]	0.10 (57)
LC	LC	1	61	12/47 (25.5)	0/14 (0)	7.81 [0.49, 124.25]	N/E (N/E)
LLETZ	LLETZ	2	3179	75/809 (9.3)	117/2370 (4.9)	2.85 [1.06, 7.69]	0.05 (73)
**Cone Depth ≥ 20mm**	**Cone Depth = 15-16 to 19-20mm**						
All Treatment types	All Treatment types	3	1560	87/851 (10.2)	55/709 (7.8)	1.46 [0.95, 2.23]	0.33 (11)
LC	LC	1	61	12/42 (28.6)	2/19 (10.5)	2.71 [0.67, 10.96]	N/E (N/E)
LLETZ	LLETZ	2	1499	75/809 (9.3)	53/690 (7.7)	1.40 [0.84, 2.36]	0.26 (22)
**Cone Depth = 11-13 to 15-16mm**	**Cone Depth ≤ 10-12mm**						
All Treatment types	All Treatment types	3	2600	75/1359 (5.5)	76/1241 (6.1)	0.92 [0.67, 1.25]	0.48 (0)

Comparison Group 1	Comparison Group 2	Studies	Total N	Treated n/N (%)	Untreated n/N (%)	Effect Estimate RR (95% CI)	Heterogeneityp -value (I^2%)
**Cone Depth**							
LLETZ	LLETZ	2	2370	57/1245 (4.6)	62/1125 (5.5)	0.83 [0.58, 1.17]	0.97 (0)
Excisional Treatment NOS	Excisional Treatment NOS	1	230	18/114 (15.8)	14/116 (12.1)	1.31 [0.68, 2.50]	N/E (N/E)
**Cone Depth = 15-16 to 19-20mm**	**Cone Depth ≤ 10-12mm**						
All Treatment types	All Treatment types	2	1815	53/690 (7.7)	62/1125 (5.5)	1.43 [1.00, 2.04]	0.53 (0)
LLETZ	LLETZ	2	1815	53/690 (7.7)	62/1125 (5.5)	1.43 [1.00, 2.04]	0.53 (0)
**Cone Depth = 15-16 to 19-20mm**	**Cone Depth ≤ 15mm**						
All Treatment types	All Treatment types	3	3093	55/709 (7.8)	117/2384 (4.9)	1.62 [1.18, 2.20]	0.66 (0)
LC	LC	1	33	2/19 (10.5)	0/14 (0)	3.75 [0.19, 72.49]	N/E (N/E)
LLETZ	LLETZ	2	3060	53/690 (7.7)	117/2370 (4.9)	1.60 [1.17, 2.19]	0.48 (0)
**Cone Volume**							
**Cone Volume ≥ 3-4cc**	**Cone Volume ≤ 3-4cc**						
All Treatment types	All Treatment types	1	278	9/60 (15.0)	16/218 (7.3)	2.04 [0.95, 4.39]	N/E (N/E)
LLETZ	LLETZ	1	278	9/60 (15.0)	16/218 (7.3)	2.04 [0.95, 4.39]	N/E (N/E)
**Cone Volume ≥ 6cc**	**Cone Volume ≤ 6cc**						
All Treatment types	All Treatment types	1	278	3/6 (50.0)	22/272 (8.1)	6.18 [2.53, 15.13]	N/E (N/E)
LLETZ	LLETZ	1	278	3/6 (50.0)	22/272 (8.1)	6.18 [2.53, 15.13]	N/E (N/E)

*If a study had more than one comparison groups, we used external groups (external general, external untreated women that had colposcopy+/-CIN+/-biopsy, women with HSIL but no treatment) in preference to internal comparators (self-matching or pre-treatment pregnancies).

CIN: cervical intraepithelial neoplasia; CKC: cold knife conisation; CT: cryotherapy; HSIL: high-grade squamous intraepithelial lesion; LA: laser ablation; LC: laser conisation; LLETZ: large loop excision of the transformation zone; N/E: not eligible; NETZ: needle excision of the transformation zone; NOS: not otherwise specified; PTB: preterm birth; RD: radical diathermy

Table E: Preterm birth (<37 weeks) for treated women versus untreated women according to the cone depth and the comparison group used

Treated Group	Untreated Group	Studies	Total N	Treated n/N (%)	Untreated n/N (%)	Effect Estimate RR (95% CI)	Heterogeneity - p value (I^2%)
**Cone Depth**							
**Cone Depth ≤ 10-12mm**							
**All Treatment types**	Untreated External	6	1026243	271/3886 (7.0)	51295/1022357 (5.0)	1.64 [1.11, 2.42]	0.003 (72)
	Untreated Internal	2	3550	174/2348 (7.4)	99/1202 (8.2)	0.90 [0.71, 1.14]	0.86 (0)
	Untreated Colposcopy+/-Biopsy	4	43145	249/3548 (7.0)	1966/39597 (5.0)	1.11 [0.85, 1.43]	0.09 (54)
**Cone Depth ≥ 10-12mm**							
**All Treatment types**	Untreated External	6	1027812	511/5455 (9.4)	51295/1022357 (5.0)	1.96 [1.66, 2.32]	0.14 (40)
	Untreated Internal	2	3944	321/2742 (11.7)	99/1202 (8.2)	2.05 [0.56, 7.48]	0.16 (50)
	Untreated Colposcopy+/-Biopsy	4	45275	544/5678 (9.6)	1966/39597 (5.0)	1.52 [1.37, 1.68]	0.36 (6)
**Cone Depth ≤ 15-17mm**							
**All Treatment types**	Untreated External	2	513145	101/2154 (4.7)	17113/510991 (3.3)	1.40 [1.16, 1.70]	0.61 (0)
	Untreated Colposcopy+/-Biopsy	3	34934	149/2600 (5.7)	1380/32334 (4.3)	1.17 [0.98, 1.39]	0.42 (0)
**Cone Depth ≥ 15-17mm**							
**All Treatment types**	Untreated External	2	512503	133/1512 (8.8)	17113/510991 (3.3)	3.04 [1.62, 5.73]	0.12 (59)
	Untreated Colposcopy+/-Biopsy	3	33934	153/1600 (9.6)	1380/32334 (4.3)	2.30 [1.57, 3.35]	0.09 (59)
**Cone Depth ≤ 20mm**							
**All Treatment types**	Untreated External	2	513814	152/2823 (5.4)	17113/510991 (3.3)	1.60 [1.37, 1.87]	0.79 (0)
	Untreated Colposcopy+/-Biopsy	2	34968	172/3060 (5.6)	1328/31908 (4.2)	1.52 [0.92, 2.51]	0.14 (54)
**Cone Depth ≥ 20mm**							
**All treatment types**	Untreated External	2	511834	84/843 (10/0)	17113/510991 (3.3)	3.63 [1.67, 7.90]	0.07 (69)
	Untreated Colposcopy+/-Biopsy	2	32717	75/809 (9.3)	1328/31908 (4.2)	4.32 [0.93, 20.03]	0.01 (87)
**Cone Depth = 10/13 to 15/16mm**							
**All Treatment types**	Untreated External	1	511959	49/1118 (4.4)	17106/510841 (3.3)	1.31 [0.99, 1.72]	N/E (N/E)
	Untreated Colposcopy+/-Biopsy	3	33693	75/1359 (5.5)	1380/32334 (4.3)	1.14 [0.90, 1.44]	0.49 (0)
**Cone Depth = 15-16 to 19-20mm**							
**All Treatment types**	Untreated External	2	511660	49/669 (7.3)	17113/510991 (3.3)	2.16 [1.65, 2.84]	0.96 (0)
	Untreated Colposcopy+/-Biopsy	2	32598	53/690 (7.7)	1328/31908 (4.2)	2.38 [1.04, 5.42]	0.08 (66)

RR: relative risk

Table F: Other maternal outcomes comparing cervical treatment techniques to no treatment*.

Maternal Outcomes	Studies	Total N	Treated n/N (%)	Untreated n/N (%)	Effect Estimate RR (95% CI)	Heterogeneity p-value (I^2%)
sPTB (<37w)						
All Treatment types	14	1024731	1181/16849 (7.0)	37257/1007882 (3.7)	1.76 [1.47, 2.11]	<0.00001 (76)
CKC	3	7320	22/154 (14.3)	291/7166 (4.1)	3.53 [2.05, 6.05]	0.38 (0)
LC	2	222	7/112 (6.3)	7/110 (6.4)	1.40 [0.51, 3.81]	0.70 (0)
NETZ	1	7399	17/71 (23.9)	301/7328 (4.1)	5.83 [3.80, 8.95]	N/E (N/E)
LLETZ	11	773123	798/10890 (7.3)	25998/762233 (3.4)	1.60 [1.22, 2.08]	<0.00001 (77)
LA	1	356	8/208 (3.8)	6/148 (4.1)	0.95 [0.34, 2.68]	N/E (N/E)
CT	1	58	1/36 (2.8)	0/22 (0)	1.86 [0.08, 43.87]	N/E (N/E)
Excisional Treatment NOS	2	95985	115/1115 (10.3)	5453/94870 (5.7)	1.70 [1.17, 2.46]	0.29 (9)
Ablative Treatment NOS	2	134720	121/2312 (5.2)	5071/132408 (3.8)	1.42 [1.20, 1.70]	0.51 (0)
Treatment NOS	1	5548	92/1951 (4.7)	130/3597 (3.6)	1.30 [1.00, 1.69]	N/E (N/E)
sPTB (<34/32w)						
All Treatment types	7	655675	225/12486 (1.8)	3787/643189 (0.6)	2.63 [1.91, 3.62]	0.01 (58)
CKC	2	6990	2/88 (2.3)	47/6902 (0.7)	4.38 [1.08, 17.65]	N/E (N/E)
NETZ	1	7399	5/71 (7.0)	49/7328 (0.7)	10.53 [4.33, 25.65]	N/E (N/E)
LLETZ	6	530985	197/10176 (1.9)	3113/520809 (0.6)	2.37 [1.82, 3.08]	0.16 (37)
CT	1	58	1/36 (2.8)	0/22 (0)	1.86 [0.08, 43.87]	N/E (N/E)
Excisional Treatment NOS	1	264	3/88 (3.4)	0/176 (0)	13.92 [0.73, 266.6]	N/E (N/E)
Ablative Treatment NOS	1	109979	17/2027 (0.8)	578/107952 (0.5)	1.57 [0.97, 2.53]	N/E (N/E)
sPTB (<28w)						
All Treatment types	2	626670	65/10917 (0.6)	1523/615753 (0.2)	3.18 [1.64, 6.16]	0.02 (68)
CKC	1	6956	1/67 (1.5)	19/6889 (0.3)	5.41 [0.74,39.84]	N/E (N/E)
NETZ	1	7399	3/71 (4.2)	21/7328 (0.3)	14.74 [4.5, 48.32]	N/E (N/E)
LLETZ	2	502336	55/8752 (0.6)	1221/493584 (0.2)	2.57 [1.96, 3.36]	0.66 (0)
Ablative Treatment NOS	1	109979	6/2027(0.3)	262/107952 (0.2)	1.22 [0.54, 2.74]	N/E (N/E)
**Threatened PTB**						
All Treatment types	5	903	31/340 (9.1)	18/563 (3.2)	2.44 [1.37, 4.33]	0.43 (0)
CKC	1	126	5/47 (10.6)	6/79 (7.6)	1.40 [0.45, 4.34]	N/E (N/E)
LC	1	112	7/53 (13.2)	5/59 (8.5)	1.56 [0.53, 4.62]	N/E (N/E)
LLETZ	1	237	4/79 (5.1)	2/158 (1.3)	4.00 [0.75, 21.37]	N/E (N/E)
Excisional Treatment NOS	2	428	15/161 (9.3)	5/267(1.9)	4.51 [1.68, 12.06]	0.52 (0)
**pPROM**						
pPROM (<37w)						
All Treatment types	21	477011	485/7903 (6.1)	15970/469108 (3.4)	2.36 [1.76, 3.17]	<0.00001 (79)
CKC	4	36733	28/194 (14.4)	930/36539 (2.5)	4.11 [2.05, 8.25]	0.12 (49)
LC	4	635	43/292 (14.7)	25/343 (7.3)	1.89 [0.97, 3.66]	0.21 (34)
NETZ	1	7279	14/71 (19.7)	161/7208 (2.2)	8.83 [5.39, 14.46]	N/E (N/E)
LLETZ	8	302974	124/2428 (5.1)	7619/300546 (2.5)	2.15 [1.48, 3.12]	0.09 (43)
LA	2	548	18/307 (5.9)	9/241 (3.7)	1.62 [0.74, 3.55]	0.64 (0)

Maternal Outcomes	Studies	Total N	Treated n/N (%)	Untreated n/N (%)	Effect Estimate RR (95% CI)	Heterogeneity p-value (I^2%)
CT	1	180	4/115 (3.5)	2/65 (3.1)	1.13 [0.21, 6.00]	N/E (N/E)
Excisional Treatment NOS	5	98372	162/2260 (7.2)	5680/96112 (5.9)	2.66 [1.13, 6.24]	<0.0001 (84)
Ablative Treatment NOS	1	24742	25/285 (8.8)	1458/24457 (6.0)	1.47 [1.01, 2.15]	N/E (N/E)
Treatment NOS	1	5548	67/1951 (3.4)	86/3597 (2.4)	1.44 [1.05, 1.97]	N/E (N/E)
pPROM (<32w)						
All Treatment types	1	72788	12/710 (1.7)	202/72078 (0.3)	8.30 [2.03, 33.98]	0.01 (78)
CKC	1	6842	1/67 (1.5)	19/6775 (0.3)	5.32 [0.72, 39.19]	N/E (N/E)
NETZ	1	7279	5/71 (7.0)	20/7208 (0.3)	25.38 [9.8, 65.74]	N/E (N/E)
LLETZ	1	58667	6/572 (1.0)	163/58095 (0.3)	3.74 [1.66, 8.41]	N/E (N/E)
pPROM (<28w)						
All Treatment types	1	72788	4/710 (0.6)	70/72078 (0.1)	9.09 [1.04, 7.18]	0.03 (72)
CKC	1	6842	0/67 (0)	7/6775 (0.1)	6.64 [0.38, 115.2]	N/E (N/E)
NETZ	1	7279	3/71 (4.2)	7/7208 (0.1)	43.51 [11.48, 164.9]	N/E (N/E)
LLETZ	1	58667	1/572 (0.2)	56/58095 (0.1)	1.81 [0.25, 13.08]	N/E (N/E)
**Chorioamnionitis**						
All Treatment types	4	29198	11/314 (3.5)	316/28884 (1.1)	3.43 [1.36, 8.64]	0.74 (0)
CKC	1	28531	2/76 (2.6)	313/28455 (1.1)	2.39 [0.61, 9.43]	N/E (N/E)
LC	1	112	1/53 (1.9)	0/59 (0)	3.33 [0.14, 80.11]	N/E (N/E)
LLETZ	1	237	5/79 (6.3)	1/158 (0.6)	10.00 [1.19, 84.15]	N/E (N/E)
Excisional Treatment NOS	1	318	3/106 (2.8)	2/212 (0.9)	3.00 [0.51, 17.68]	N/E (N/E)
**Mode of Delivery**						
Caeserean Section						
All Treatment types	36	272360	1784/8942 (20.0)	46929/263418 (17.8)	1.06 [0.98, 1.14]	0.15 (19)
CKC	6	30462	54/308 (17.5)	3698/30154 (12.3)	1.24 [0.91, 1.68]	0.36 (9)
LC	5	1038	57/445 (12.8)	63/593 (10.6)	1.38 [0.90, 2.11]	0.23 (29)
LLETZ	14	5436	509/2363 (21.5)	672/3073 (21.9)	1.04 [0.94, 1.15]	0.71 (0)
LA	4	1258	50/510 (9.8)	86/748 (11.5)	0.86 [0.61, 1.20]	0.62 (0)
CT	2	238	24/151 (15.9)	5/87 (5.7)	2.47 [1.02, 6.01]	0.32 (0)
Excisional Treatment NOS	8	203262	622/2713 (22.9)	36670/200549 (18.3)	1.06 [0.90, 1.25]	0.06 (49)
Ablative Treatment NOS	2	24848	71/366 (19.4)	5103/24482 (20.8)	1.38 [0.42, 4.58]	0.17 (48)
Treatment NOS	2	5818	397/2086 (19.0)	632/3732 (16.9)	1.03 [0.78, 1.35]	0.13 (56)
Instrumental Deliveries (ventouse/forceps)						
All Treatment types	16	9588	484/3773 (12.8)	793/815 (13.6)	0.97 [0.88, 1.08]	0.72 (0)
CKC	2	454	10/128 (7.8)	24/326 (7.4)	1.33 [0.66, 2.70]	0.40 (0)
LC	2	668	21/306 (6.9)	22/362 (6.1)	1.16 [0.65, 2.07]	0.66 (0)
LLETZ	6	1418	85/689 (12.3)	98/729 (13..4)	0.89 [0.68, 1.17]	0.70 (0)
LA	3	550	39/274 (14.2)	42/276 (15.2)	0.94 [0.62, 1.41]	0.37 (0)
Excisional Treatment NOS	3	950	33/425 (7.8)	68/525 (13.0)	0.71 [0.46, 1.10]	0.32 (11)
Treatment NOS	1	5548	296/1951 (15.2)	539/3597 (15.0)	1.01 [0.89, 1.15]	N/E (N/E)
**Length of Labour**						

Maternal Outcomes	Studies	Total N	Treated n/N (%)	Untreated n/N (%)	Effect Estimate RR (95% CI)	Heterogeneity p-value (I^2%)
Precipitous Labour (<2h)						
All Treatment types	5	1059	34/397 (8.6)	43/662 (6.5)	1.26 [0.80, 1.96]	1.00 (0)
CKC	2	289	5/71 (7.0)	15/218 (6.9)	1.24 [0.47, 3.27]	N/E (N/E)
LLETZ	4	770	29/326 (8.9)	28/444 (6.3)	1.26 [0.76, 2.08]	1.00 (0)
Prolonged Labour (>12 h)						
All Treatment types	7	1854	76/859 (8.8)	75/995 (7.5)	1.25 [0.92, 1.69]	0.59 (0)
CKC	2	325	8/91 (8.8)	15/234 (6.4)	1.99 [0.89, 4.45]	N/E (N/E)
LC	1	500	11/50 (4.4)	12/50 (4.8)	0.92 [0.41, 2.04]	N/E (N/E)
LLETZ	4	673	22/341 (6.5)	23/332 (6.9)	0.96 [0.55, 1.70]	0.48 (0)
LA	2	356	35/177 (19.8)	25/179 (14.0)	1.41 [0.88, 2.26]	0.60 (0)
**Induction of Labour**						
All Treatment types	11	4668	477/1971 (24.2)	638/2697 (23.7)	1.01 [0.89, 1.15]	0.34 (10)
CKC	2	137	14/73 (19.2)	10/64(15.6)	1.11 [0.54, 2.29]	0.75 (0)
LLETZ	8	4056	421/1712 (24.6)	551/2344 (23.5)	0.99 [0.82, 1.20]	0.13 (38)
CT	1	58	6/36 (16.7)	6/22 (27.3)	0.61 [0.22, 1.66]	N/E (N/E)
Excisional Treatment NOS	2	417	36/150 (24.0)	71/267 (26.6)	0.90 [0.64, 1.28]	0.79 (0)
**Oxytocin Use**						
All Treatment types	6	2006	166/978 (17.0)	180/1028 (17.5)	0.90 [0.64, 1.26]	0.04 (58)
CKC	1	103	19/52 (36.5)	19/51 (37.3)	0.98 [0.59, 1.63]	N/E (N/E)
LLETZ	4	1804	131/882 (14.9)	144/922 (15.6)	0.76 [0.43, 1.34]	0.01 (74)
Excisional Treatment NOS	1	99	16/44 (36.4)	17/55 (30.9)	1.18 [0.67, 2.05]	N/E (N/E)
**Haemorrhage**						
Antepartum Haemorrhage						
All Treatment types	4	1245	24/502 (4.8)	21/743 (2.8)	1.11 [0.40, 3.12]	0.03 (59)
CKC	1	34	4/21 (19.0)	2/13 (15.4)	1.24 [0.26, 5.83]	N/E (N/E)
LC	1	168	4/56 (7.1)	0/112 (0.0)	17.84 [0.98, 325.7]	N/E (N/E)
LLETZ	2	277	10/153 (6.5)	15/124 (12.1)	0.52 [0.16, 1.67]	0.15 (53)
LA	1	708	4/236 (1.7)	1/472 (0.2)	8.00 [0.90, 71.18]	N/E (N/E)
CT	1	58	2/36 (5.6)	3/22 (13.6)	0.41 [0.07, 2.25]	N/E (N/E)
Postpartum Haemorrhage (>600ml)						
All Treatment types	1	149	14/75 (18.7)	3/74 (4.1)	4.60 [1.38, 15.36]	N/E (N/E)
CKC	1	149	14/75 (18.7)	3/74 (4.1)	4.60 [1.38, 15.36]	N/E (N/E)
Massive Obstetric Haemorrhage (>1000ml)						
All Treatment types	1	149	4/75 (5.3)	1/74 (1.4)	3.95 [0.45, 34.48]	N/E (N/E)
CKC	1	149	4/75 (5.3)	1/74 (1.4)	3.95 [0.45, 34.48]	N/E (N/E)
**Analgesia**						
Epidural Use						
All Treatment types	5	105488	87/442 (19.7)	23205/105046 (22.1)	1.02 [0.68, 1.53]	0.02 (64)
LLETZ	4	818	66/389 (17.0)	85/429 (19.8)	0.86 [0.64, 1.16]	0.86 (0)
Excisional Treatment NOS	1	104670	21/53 (9.6)	23120/104617 (22.1)	1.79 [1.29, 2.50]	N/E (N/E)

Maternal Outcomes	Studies	Total N	Treated n/N (%)	Untreated n/N (%)	Effect Estimate RR (95% CI)	Heterogeneity p-value (I^2%)
Pethidine Use						
All Treatment types	2	394	61/197 (31.0)	64/197 (32.5)	0.94 [0.72, 1.24]	0.62 (0)
LLETZ	2	394	61/197 (31.0)	64/197 (32.5)	0.94 [0.72, 1.24]	0.62 (0)
Analgesia use NOS						
All Treatment types	1	103	17/52 (32.7)	15/51 (29.4)	1.11 [0.62, 1.98]	N/E (N/E)
CKC	1	103	17/52 (32.7)	15/51 (29.4)	1.11 [0.62, 1.98]	N/E (N/E)
**Cervical cerclage**						
All Treatment types	8	141300	97/2416 (4.0)	932/138884 (0.7)	14.29 [2.85, 71.65]	<0.00001 (93)
CKC	3	30744	41/246 (16.7)	71/30498 (0.2)	31.42 [2.32, 426.22]	0.07 (62)
LC	1	112	6/53 (11.3)	1/59 (1.7)	6.68 [0.83, 53.69]	N/E (N/E)
LLETZ	1	56	5/28 (17.9)	0/28 (0)	11.00 [0.64, 189.96]	N/E (N/E)
Excisional Treatment NOS	2	104840	18/138 (13.0)	837/104702 (0.8)	42.45 [28.99, 62.16]	N/E (N/E)
Treatment NOS	1	5548	27/1951 (1.4)	23/3597 (0.6)	2.16 [1.24, 3.76]	N/E (N/E)
**Cervical stenosis**						
All Treatment types	2	680	2/365 (0.5)	0/315 (0.0)	2.26 [0.24, 21.59]	0.81 (0)
LC	1	500	1/250 (0.4)	0/250 (0.0)	3.00 [0.12, 73.29]	N/E (N/E)
CT	1	180	1/115 (0.9)	0/65 (0.0)	1.71 [0.07, 41.31]	N/E (N/E)

*If a study had more than one comparison groups, we used external groups (external general, external untreated women that had colposcopy+/-CIN+/-biopsy, women with HSIL but no treatment) in preference to internal comparators (self-matching or pre-treatment pregnancies).

CKC: cold knife conisation; CT: cryotherapy; g: grams; LA: laser ablation; LBW: low birth weight; LC: laser conisation; LLETZ: large loop excision of the transformation zone; min: minute; N/E: not eligible; NETZ: needle excision of the transformation zone; NICU: neonatal intensive care unit; NOS: not otherwise specified; pPROM: preterm premature rupture of membranes PTB: preterm birth; sPTB: spontaneous preterm birth; w: weeks

Table G: Neonatal outcomes comparing cervical treatment techniques to no treatment*.

Neonatal Outcomes	Studies	Total N	Treated n/N (%)	Untreated n/N (%)	Effect Estimate RR (95% CI)	Heterogeneity p-value (I^2%)
**Birth weight**						
LBW (<2500g)						
All Treatment types	30	1348206	1542/19489 (7.9)	48632/1328717 (3.7)	1.81 [1.58, 2.07]	<0.00001 (63)
CKC	5	30304	49/246 (19.9)	2308/30058 (7.7)	2.51 [1.78, 3.53]	0.79 (0)
LC	4	786	29/336 (8.6)	30/450 (6.7)	1.76 [0.72, 4.35]	0.04 (63)
LLETZ	12	3357	157/1605 (9.8)	83/1752 (4.7)	2.11 [1.51, 2.94]	0.13 (32)
LA	4	1104	29/421 (6.9)	42/683 (6.1)	1.07 [0.59, 1.92]	0.29 (20)
CT	1	58	6/36 (16.7)	1/22 (4.5)	3.67 [0.47, 28.47]	N/E (N/E)
Excisional Treatment NOS	10	823648	840/10416 (8.1)	29739/813232 (3.7)	2.01 [1.62, 2.49]	<0.00001 (78)
Ablative Treatment NOS	4	483402	220/4478 (4.9)	16140/478924 (3.4)	1.36 [1.19, 1.55]	0.88 (0)
Treatment NOS	1	5547	212/1951 (10.9)	289/3596 (8.0)	1.35 [1.14, 1.60]	N/E (N/E)
LBW (<2000g)						
All Treatment types	3	74981	50/1053 (4.7)	788/73928 (1.1)	2.49 [0.97, 6.36]	0.01 (72)
LC	1	181	7/51 (13.7)	4/130 (3.1)	4.46 [1.36, 14.59]	N/E (N/E)
LA	2	772	7/256 (2.7)	15/516 (2.9)	0.95 [0.39, 2.29]	0.89 (0)
Excisional Treatment NOS	1	74028	36/746 (4.8)	769/73282 (1.0)	4.60 [3.32, 6.37]	N/E (N/E)
LBW (<1500g)						
All Treatment types	5	76836	39/1977 (2.0)	390/74859 (0.5)	3.00 [1.54, 5.85]	0.24 (26)
LC	1	181	5/51 (9.8)	1/130 (0.8)	12.75 [1.53, 106.44]	N/E (N/E)
LLETZ	1	378	3/189 (1.6)	0/189 (0)	7.00 [0.36, 134.59]	N/E (N/E)
LA	2	772	2/256 (0.8)	7/516 (1.4)	0.68 [0.16, 2.80]	0.97 (0)
Excisional Treatment NOS	2	75505	29/1481 (2.0)	382/74024 (0.5)	3.34 [2.02, 5.54]	0.61 (0)
LBW (<1000g)						
All Treatment types	2	2185	11/971 (1.1)	4/1214 (0.3)	2.09 [0.06, 74.71]	0.05 (75)
LA	1	708	0/236 (0)	3/472 (0.6)	0.29 [0.01, 5.50]	N/E (N/E)
Excisional Treatment NOS	1	1477	11/735 (1.5)	1/742 (0.1)	11.10 [1.44, 85.79]	N/E (N/E)
**NICU Admission**						
All Treatment types	8	2557	155/1226 (12.6)	119/1331 (8.9)	1.45 [1.16, 1.81]	0.73 (0)
CKC	2	71	6/35 (17.1)	6/36 (16.7)	1.40 [0.52, 3.75]	0.50 (0)
LLETZ	5	1994	110/991 (11.1)	81/1003 (8.1)	1.42 [1.01, 1.99]	0.36 (8)
CT	1	58	4/36 (11.1)	1/22 (4.5)	2.44 [0.29, 20.49]	N/E (N/E)
Excisional Treatment NOS	2	434	35/164 (21.3)	31/270 (11.5)	1.76 [1.13, 2.75]	0.85 (0)
**Perinatal Mortality**						

Neonatal Outcomes	Studies	Total N	Treated n/N (%)	Untreated n/N (%)	Effect Estimate RR (95% CI)	Heterogeneity p-value (I^2%)
Perinatal mortality overall						
All Treatment types	23	1659433	149/15817 (0.9)	11687/1643616 (0.7)	1.51 [1.13, 2.03]	0.04 (36)
CKC	7	50588	16/573 (2.8)	945/50015 (1.9)	1.46 [0.83, 2.57]	0.93 (0)
LC	3	906	6/376 (1.6)	5/530 (0.9)	1.89 [0.26, 13.87]	0.10 (63)
NETZ	1	7399	3/71 (4.2)	31/7328 (0.4)	9.99 [3.13, 31.92]	N/E (N/E)
LLETZ	7	302271	17/1925 (0.9)	2430/300346 (0.8)	1.53 [0.88, 2.67]	0.93 (0)
LA	2	258	1/117 (0.9)	0/141 (0)	3.00 [0.12, 72.74]	N/E (N/E)
CT	2	238	0/151 (0)	1/87 (1.1)	0.19 [0.01, 4.59]	N/E (N/E)
Excisional Treatment NOS	5	820028	63/6792 (0.9)	5427/813236 (0.7)	1.85 [1.02, 3.36]	0.08 (56)
Ablative Treatment NOS	2	472197	16/3861 (0.4)	2798/468336 (0.6)	0.69 [0.42, 1.13]	0.77 (0)
Treatment NOS	1	5548	27/1951 (1.4)	50/3597 (1.4)	1.00 [0.63, 1.58]	N/E (N/E)
Perinatal Mortality (<37w)						
All Treatment types	1	73992	6/710 (0.8)	98/73282 (0.1)	9.40 [2.01, 43.89]	0.06 (65)
CKC	1	6956	0/67 (0)	9/6889 (0.1)	5.33 [0.31, 90.71]	N/E (N/E)
NETZ	1	7399	3/71 (4.2)	10/7328 (0.1)	30.96 [8.71, 110.13]	N/E (N/E)
LLETZ	1	59637	3/572 (0.5)	79/59065 (0.1)	3.92 [1.24, 12.38]	N/E (N/E)
Perinatal Mortality (<32w)						
All Treatment types	1	73992	6/710 (0.8)	71/73282 (0.1)	12.77 [2.51, 64.99]	0.05 (67)
CKC	1	6956	0/67 (0)	7/6889 (0.1)	6.75 [0.39, 117.10]	N/E (N/E)
NETZ	1	7399	3/71 (4.2)	7/7328 (0.1)	44.23 [11.67, 167.61]	N/E (N/E)
LLETZ	1	59637	3/572 (0.5)	57/59065 (0.1)	5.43 [1.71, 17.30]	N/E (N/E)
Perinatal Mortality (<28w)						
All Treatment types	1	73992	5/710 (0.7)	57/73282 (0.1)	13.76 [2.37, 79.89]	0.05 (67)
CKC	1	6956	0/67 (0)	5/6889 (0.1)	9.21 [0.51, 164.95]	N/E (N/E)
NETZ	1	7399	3/71 (4.2)	6/7328 (0.1)	51.61 [13.17, 202.29]	N/E (N/E)
LLETZ	1	59637	2/572 (0.3)	46/59065 (0.1)	4.49 [1.09, 18.45]	N/E (N/E)
**Stillbirth**						
All Treatment types	12	249855	28/3920 (0.7)	1376/245935 (0.6)	0.98 [0.63, 1.52]	0.80 (0)
CKC	3	935	5/325 (1.5)	5/610 (0.8)	1.61 [0.48, 5.40]	0.66 (0)
LC	2	725	1/325 (0.3)	3/400 (0.8)	0.33 [0.03, 3.18]	N/E (N/E)
LLETZ	4	242473	7/1244 (0.6)	1332/241229 (0.6)	1.42 [0.62, 3.26]	0.84 (0)
LA	1	64	0/20 (0)	0/44 (0)	N/E	N/E (N/E)
Treatment NOS	1	5548	15/1951 (0.8)	36/3597 (1.0)	0.77 [0.42, 1.40]	N/E (N/E)
Excisional Treatment NOS	1	110	0/55 (0)	0/55 (0)	N/E	N/E (N/E)

Neonatal Outcomes	Studies	Total N	Treated n/N (%)	Untreated n/N (%)	Effect Estimate RR (95% CI)	Heterogeneity p-value (I^2%)
**Apgar score**						
Apgar score (≤5)(1min)						
All Treatment types	1	225	2/75 (2.7)	7/150 (4.7)	0.57 [0.12, 2.68]	N/E (N/E)
LC	1	225	2/75 (2.7)	7/150 (4.7)	0.57 [0.12, 2.68]	N/E (N/E)
Apgar score (<7)(1min)						
All Treatment types	1	152	2/84 (2.4)	3/68 (4.4)	0.63 [0.07, 5.71]	0.24 (28)
LLETZ	1	87	0/48 (0)	2/39 (5.1)	0.16 [0.01, 3.30]	N/E (N/E)
CKC	1	65	2/36 (5.6)	1/29 (3.4)	1.61 [0.15, 16.90]	N/E (N/E)
Apgar score (<7)(5min)						
All Treatment types	2	297	4/159 (2.5)	3/138 (2.2)	0.82 [0.19, 3.59]	0.80 (0)
CKC	1	32	0/20 (0)	0/12 (0)	N/E	N/E (N/E)
LLETZ	1	120	3/74 (4.1)	2/46 (4.3)	0.93 [0.16, 5.37]	N/E (N/E)
CT	1	58	1/36 (2.8)	1/22 (4.5)	0.61 [0.04, 9.28]	N/E (N/E)
Excisional Treatment NOS	1	87	0/29 (0)	0/58 (0)	N/E	N/E (N/E)

*If a study had more than one comparison groups, we used external groups (external general, external untreated women that had colposcopy+/-CIN+/-biopsy, women with HSIL but no treatment) in preference to internal comparators (self-matching or pre-treatment pregnancies).

CKC: cold knife conisation; CT: cryotherapy; g: grams; LA: laser ablation; LBW: low birth weight; LC: laser conisation; LLETZ: large loop excision of the transformation zone; min: minute; N/E: not eligible; NETZ: needle excision of the transformation zone; NICU: neonatal intensive care unit; NOS: not otherwise specified; w: weeks
