# Supplementary figures and images for: Adverse obstetric outcomes after local treatment for cervical preinvasive and early invasive disease according to cone depth: systematic review and meta-analysis
Source: BMJ. 2016 Jul 28;354:i3633. doi: 10.1136/bmj.i3633 (PMC4964801; doi:10.1136/bmj.i3633)

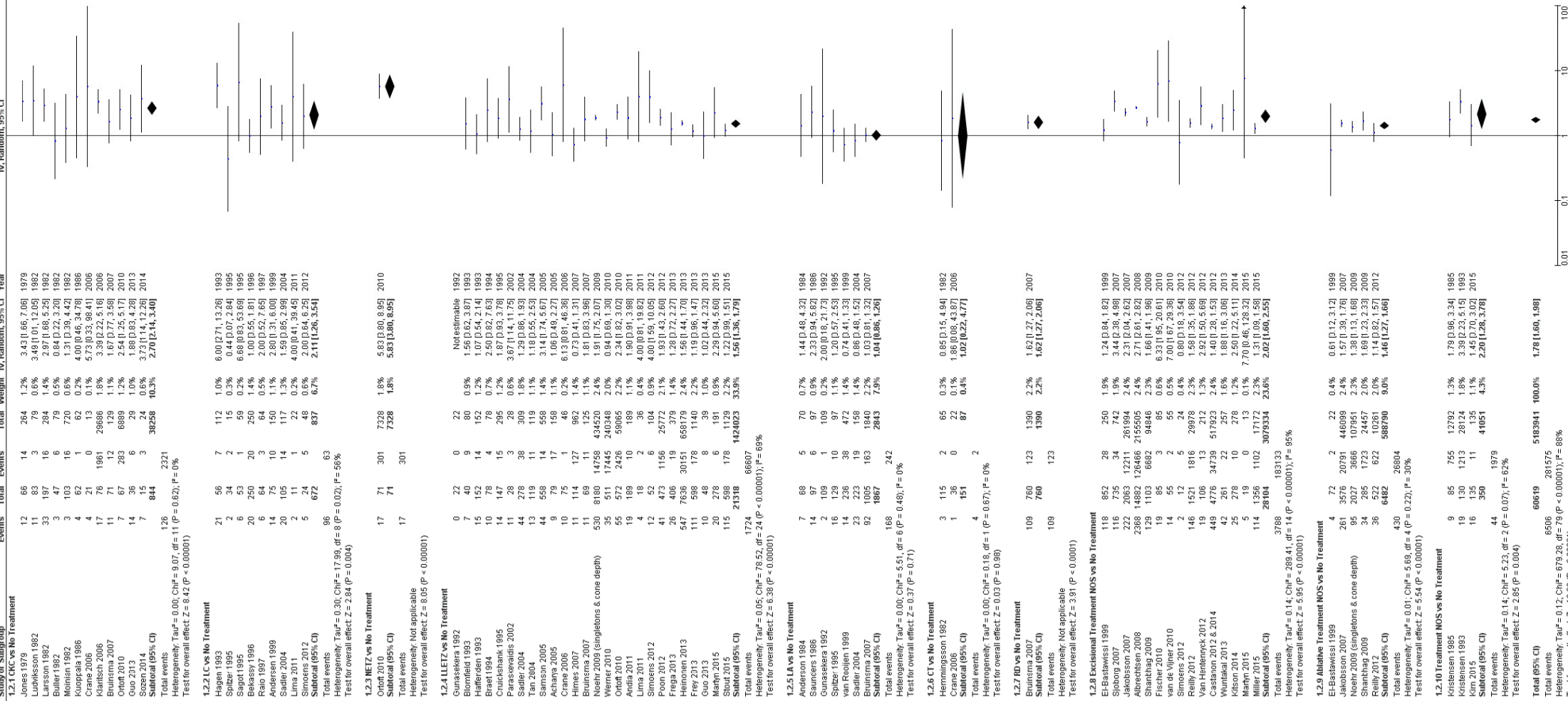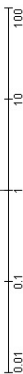

0.01 0.1 1 10 100  
More Harm Untreated Group More Harm Treated Group

Supplement: Supplementary file 3 — Appendix 3: Forest plot for all treatment techniques [file kyrm031600.ww3_default.pdf]
